# Analysis of Tryptophan and Its Main Metabolite Kynurenine and the Risk of Multiple Cancers Based on the Bidirectional Mendelian Randomization Analysis

**DOI:** 10.3389/fonc.2022.852718

**Published:** 2022-04-14

**Authors:** Ran Li, Xuanyang Wang, Yuntao Zhang, Xiaoqing Xu, Lulu Wang, Chunbo Wei, Lin Liu, Ziqi Wang, Ying Li

**Affiliations:** Department of Nutrition and Food Hygiene, the National Key Discipline, School of Public Health, Harbin Medical University, Harbin, China

**Keywords:** tryptophan, kynurenine, multiple cancers, causation, Mendelian randomization analysis

## Abstract

**Background:**

Tryptophan and its metabolites have been found related to various cancers, but the direction of this relationship is still unclear. The purpose of this study is to explore the causal associations of tryptophan and kynurenine with multiple cancers based on the bidirectional Mendelian randomization analysis.

**Methods:**

The data of a genome-wide association study meta-analysis on 7,824 individuals was used to explore the genetic variants strongly associated with tryptophan and kynurenine. Genetic instruments of four specific cancers were obtained from available summary-level data of 323,590 European participants. Bidirectional Mendelian randomization analysis was conducted to examine possible causality. Sensitivity analysis was performed to test heterogeneity and horizontal pleiotropy. COX regression analysis was conducted to explore associations between dietary tryptophan and cancer mortality in NHANES 1988-1994.

**Results:**

No evidence of any causal association of tryptophan and kynurenine with the risk of four specific cancers was shown, except for weak correlations were suggested between lung or prostate cancer and kynurenine. Multiple sensitivity analyses generated similar results. Our findings from COX regression analysis were consistent with the above results.

**Conclusions:**

Our study did not find any causal relationship between tryptophan and kynurenine and multiple cancers. The associations still need further research.

## 1 Introduction

As an essential amino acid for the human body, tryptophan and a series of intermediate products of its metabolic pathways (serotonin pathway, kynurenine pathway and indole pathway) have become the therapeutic targets for depression, schizophrenia, neurodegenerative diseases, autoimmune diseases and cancers (1), such as some of the main rate-limiting enzymes (2) tryptophan-2,3-dioxygenase (TDO), kynurenine monooxygenase (KMO), indoleamine-2,3-dioxygenase 1 (IDO1) and indoleamine-2,3-dioxygenase 2 (IDO2).

Most existing studies have shown that tryptophan metabolic pathways including tryptophan degradation, kynurenine synthesis and overactivation of some major rate-limiting enzymes can promote tumor progression by inhibiting anti-tumor immune responses, limiting tumor immune infiltration and enhancing the malignant characteristics of cancer cells (3). Case-control studies have suggested that tryptophan metabolism pathways play a role in regulating regulatory T cells and in the infiltration of immune cells in cancer (4). The inhibitory effect of tryptophan metabolism pathways on immune cells is believed to be achieved by increasing the immunosuppressive catabolites of tryptophan and reducing tryptophan (5). *In vitro* experiments have found that higher levels of kynurenine, the main metabolite of tryptophan, were suggested to increase the proliferation and migration ability of cancer cells and help tumors avoid immune surveillance by reducing the activity of natural killer cells, dendritic cells or proliferating T cells (6). In addition, animal experiments have also indicated that enzymes involved in tryptophan metabolism are expressed in a variety of cancers. IDO1 is expressed in about 58% of human tumors and is related to the adverse clinical outcomes of various cancers, including melanoma, gynecological cancer and hematological malignancies (7).

However, the above associations have not been replicated in other studies. Some studies suggested that the immunomodulatory properties of the tryptophan metabolism pathways were mainly results of the influence of metabolites of the kynurenine pathway, rather than results of the reduction of tryptophan (8). Although the levels of systemic tryptophan in patients with lung cancer (9), malignant glioma (10), malignant melanoma (11), rectal cancer (12) and gynecological-related cancers (13) showed a downward trend, the elevation of the metabolites of the kynurenine pathway in the blood was rarely observed, which may be due to the small local changes of kynurenine and its downstream metabolites in tumor microenvironment.

Moreover, the link between tryptophan metabolism pathways and cancer has prompted a lot of researches on treatments targeting the kynurenine pathway, especially by inhibiting the key enzymes including TDO, IDO1 and KMO (1). Although current clinical trials have found some of these key enzyme inhibitors achieved the expected effects in early cancer immunotherapy, the results of phase III trials were negative. Given the mixed results, the unclear causal relationship between tryptophan and its main metabolite kynurenine and cancer still needs to be clarified.

In general, various cancers have been reported to be related to the changes of tryptophan and its metabolites in human body, such as lung cancer (9), breast cancer (14–16), colon cancer (17), rectal cancer (12), ovarian cancer (18), prostate cancer (19, 20), malignant glioma (10), malignant melanoma (11) and T cells leukemia (21), etc. However, no uniform convincing conclusion has been drawn so far.

Mendelian randomization (MR) is a causal inference method that uses genetic variations as exposure tools to estimate the causal influences of exposures on outcomes, based on the Mendelian Law of Independence. It overcomes the inherent confounding factors of general researches and provides reasonable temporality of causal inference (22).

In order to explore the causal associations between changes in the tryptophan metabolic pathways, including tryptophan and its main metabolite kynurenine, and the risk of site‐specific cancers such as breast, lung, prostate and ovarian cancers, a bidirectional MR analysis was conducted in this study. To evaluate the presumed causal relationship, tryptophan and kynurenine existed in either plasma or serum were considered to be exposure factors and the risk of site-specific cancers was considered to be outcome in forward MR analysis. During reverse MR analysis, the risk of breast, lung, prostate and ovarian cancer was selected as exposure factor and the plasma or serum tryptophan and kynurenine concentrations were chosen as outcomes. Throughout the bidirectional MR analysis, SNPs strongly associated with the selected exposure factors (P < 5×10^-8^) were used as genetic instruments. Meanwhile, we used NHANES 1988-1994 (NHANES III) data to analyze the association of dietary tryptophan intake and cancer mortality.

## 2 Materials and Methods

### 2.1 Bidirectional Mendelian Randomization Analysis

#### 2.1.1 Study Design Overview

**Figure 1:**
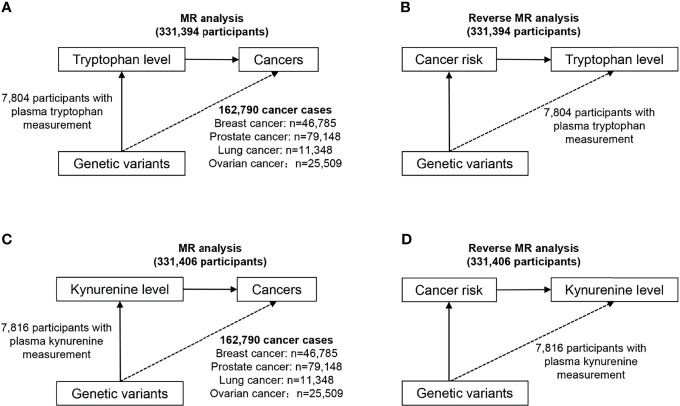
Study design overview of the bidirectional MR analysis. **(A)** Design of the MR analysis of the causal association of circulating tryptophan levels with the risk of site-specific cancers (upper left). **(B)** Design of the reverse MR analysis of the causal association of site-specific cancers with circulating tryptophan levels (upper right). **(C)** Design of the MR analysis of the causal association of circulating kynurenine levels with the risk of site-specific cancers (lower left). **(D)** Design of the reverse MR analysis of the causal association of site-specific cancers with circulating kynurenine levels (lower right).

**Figure 1** provides an overview of the participating studies and overall design of the MR analysis performed. We have identified SNPs that have a strong correlation with target exposure in published public data, and then explored whether there is a potential causal relationship between them and the corresponding outcomes. Briefly, we conducted bidirectional MR analysis twice, one to evaluate the potential causal association between tryptophan and cancer, and the other to evaluate the potential causal relationship of kynurenine and cancer.

#### 2.1.2 Selection of Genetic Instruments Strongly Associated With Tryptophan or Kynurenine

Summary statistics of a meta-analysis of Genome‐wide Association Studies (GWAS) were obtained. From this study, SNPs strongly associated with tryptophan or kynurenine, at a statistically significance level (P < 5×10^‐8^) were identified, by genotyping 7,824 adult individuals from 2 European population studies (23) (KORA‐TwinsUK studies). Then pairwise-linkage disequilibrium (LD) clumping with a clumping window of 10 MB and an r^2^ cutoff of 0.001 was applied to ensure independence among genetic instruments. To evaluate the weak instrument bias, F-statistic for each SNP was calculated. SNPs with low statistical power were removed (24) (F-statistics < 10).

To make sure the effects of SNPs on the exposure correspond to the same allele as their effects on outcome, the matching of effect alleles of each SNP between the summary statistics of the exposure and the outcome was examined using the harmonise_data function (25). Finally, we selected 18 tryptophan-related SNPs and 4 kynurenine-related SNPs as genetic instruments for the MR analysis. The details of each SNP are described in **Supplementary Table S1**.

#### 2.1.3 Selection of Genetic Instruments Strongly Associated With Breast Cancer, Lung Cancer, Prostate Cancer or Ovarian Cancer

Instrumental variables associated with breast cancer, lung cancer, prostate cancer or ovarian cancer were selected from the summary statistics of a meta-analysis of GWAS with numbers of cases ranging from 11,348 (lung cancer) to 79,148 (prostate cancer). Publicly available summary-level data for these cancers were obtained from the Breast Cancer Association Consortium (BCAC), International Lung Cancer Consortium (ILCCO), the Prostate Cancer Association Group to Investigate Cancer Associated Alterations in the Genome (PRACTICAL) and the Ovarian Cancer National Alliance (OCAC) respectively. Briefly, the BCAC and PRACTICAL consortiums aim to identify genes that are related to the risk of breast and prostate cancer by combining data from many studies. The ILCCO was established in 2004, with the goal of sharing compatible data from lung cancer epidemiology studies around the world to maximize statistical power. The OCAC consortium was founded in 2005 dedicated to foster collaborative efforts to discover and validate associations between genetic polymorphisms and the risk of ovarian cancer (18). After pairwise-linkage disequilibrium (LD) clumping and matching of coding alleles between exposure and outcome, we obtained 30 SNPs for breast cancer, 3 SNPs for lung cancer, 89 SNPs for prostate cancer and 7 SNPs for ovarian cancer. The details of each SNP are described in **Supplementary Table S2**.

#### 2.1.4 Outcome Data Sources

Publicly available summary-level data for breast, lung, prostate and ovarian cancer were obtained from the BCAC, ILCCO, PRACTICAL and OCAC using the MR-Base database. Summary-level data for tryptophan and kynurenine were obtained from the KORA-TwinsUK studies up to 7824 adult individuals of European descent (26, 27). Detailed information on the above sources are described in **Supplementary Tables S3–S5**.

#### 2.1.5 Statistical Analysis

Based on the publicly available GWAS summary statistics we retrieved, a bidirectional MR analysis was conducted. Firstly, we performed a MR analysis using the inverse-variance-weighted (IVW) method (28) as our primary MR method to assess the association between genetically predicted circulating tryptophan levels and cancers. To reduce the possibility that the genetic instruments of exposure affect the outcome independently, the following sensitivity analysis methods were chosen: the maximum likelihood method, the simple median method, the weighted median method (29) and the penalised weighted median. Secondly, we carried out a reverse MR analysis to examine the potential causal association of site-specific cancers with circulating tryptophan levels. The IVW method was also treated as the primary approach. We conducted sensitivity analysis using the MR-Egger method, the weighted median method, the simple mode method and the weighted mode method to evaluate the possible violation of the MR assumptions and the robustness of the results. MR-Egger regression (30), MR-PRESSO global test (31) and Cochran,s Q test were executed to detect the degree of heterogeneity and horizontal pleiotropy among estimates of SNPs in each analysis. The above statistical methods for the bidirectional MR analysis were also used to explore the relationship between circulating kynurenine levels and site‐specific cancers.

Statistical analysis was conducted in R 4.1.1 and MR Base platform (32) (http://app.mrbase.org/). All *P*-values were two-tailed and associations were considered statistically significant at *P* < 0.05.

### 2.2 Cox Proportional Hazards Model

#### 2.2.1 Study Population

A total of 16,678 adults met the inclusion and exclusion criteria in NHANES III were selected in our study. The NHANES III survey was conducted by the National Center for Health Statistics (NCHS) of the Centers for Disease Control and Prevention (CDC) between 1988 and 1994, which was designed to examine the health and nutritional status of the noninstitutionalized U.S. population (33). It contains two parts of data, interviews and examinations, based on demographic, socioeconomic, dietary, health-related questions, physiological measurements, laboratory tests and other information administered by highly trained medical personnel (34). All procedures were approved by CDC’s Institutional Review Board (IRB) and all study subjects provided written informed consent (35). Participants were included if they were aged 18 years and above and were excluded if they had missing values on dietary tryptophan intake and/or all-cancer mortality information (n=2,920).

#### 2.2.2 Measurements

Each participant underwent anthropometric measurements, provided a blood sample and completed a detailed questionnaire on sociodemographic, lifestyle and health-related factors (36). Dietary tryptophan intake, considered as an exposure variable in this study was estimated by a 24-hour recall methodology, collected through an automated interview with the Dietary Data Collection (DDC) system (37). Information on age (years), gender (male, female), education (years), poverty income ratio (continuous), healthy eating index score (continuous), race (Non-Hispanic White, Non-Hispanic Black, Mexican-American and other race), smoker (yes, no), drinker (yes, no), regular exercise (yes, no), diabetes (yes, no), hypertension (yes, no) and cancer (yes, no) was based on self-report during the questionnaire portion of the NHANES III survey. Body mass index (BMI) was calculated as the ratio of weight (kg) to the square of height (m). Poverty income ratio is the ratio of the midpoint of observed family income category to the official poverty threshold (38). C-reactive protein level was measured by high-sensitivity latex-enhanced nephelometry by CDC (39). Smokers were defined as adults who have smoked 100 cigarettes in their lifetime and who currently smoke cigarettes. Drinkers were defined as individuals who reported having at least 12 drinks in the last 12 months. Hypercholesterolemia was defined as a cholesterol level greater than 239 mg/dL (40).

To determine the final mortality status of every participant, multiple sources of information were utilized by NCHS, including the National Death Index (NDI), the Second Longitudinal Study of Aging (LSOA II), the Centers for Medicare and Medicaid Services (CMS) and death certificates (41). The outcome was all-cancer mortality status ascertained by NDI (42). Death due to cancer was defined as ICD-10 coding C00-C97 (43).

#### 2.2.3 Statistical Analysis

According to the analysis guidelines downloaded from NHANES III (44), mean, proportion and confidence interval (CI) of variables were calculated, considering the complex, stratified sampling design by applying weights, strata and sampling unit values to produce estimates of the U.S. population. Baseline characteristics are described by quintiles of dietary tryptophan intake separately for participants with cancer and non-cancer subjects. Continuous variables and categorical variables are described as mean (95% CI) and percentage (95% CI) respectively. General linear models (for continuous variables) and chi-square tests (for categorical variables) were conducted to assess univariate relations among different groups.

Cox proportional hazards (CPH) models were used to evaluate the association of dietary tryptophan intake with all-cancer mortality by calculating hazard ratios (HRs) and its 95% CIs. Survival time was defined as the months between NHANES III interview date and death or census date.

To check the PH assumption of the Cox regression models, a graphical method based on the Kaplan-Meier test was adopted (45). According to the present analysis, dietary tryptophan intake satisfied the PH assumption (*P* > 0.05). Potential confounders we selected were well-established or biological interest factors.

Statistical analysis was conducted in R 4.1.1 and MR Base platform (32) (http://app.mrbase.org/). All *P*-values were two-tailed and associations were considered statistically significant at *P* < 0.05.

### 2.3 Data Availability

The summary statistics for tryptophan and kynurenine GWAS by Shin et al. (23) are available at http://mips.helmholtz-muenchen.de/proj/GWAS/gwas/index.php. The breast (90), lung (91), prostate (92) and ovarian (93) cancer GWAS summary data are derived from https://gwas.mrcieu.ac.uk/. The data of NHANES III are available at https://wwwn.cdc.gov/nchs/nhanes/nhanes3/default.aspx.

## 3 Results

### 3.1 Information on the Selected SNPs and the Population Involved in the Study

General selection process was reflected by the Manhattan plots of the SNPs strongly associated with circulating tryptophan and kynurenine levels (**Supplementary Figure S1**). By drawing quantile-quantile (Q-Q) plots and calculating genomic inflation factors, the conclusion was that the selected SNPs and their corresponding traits were significantly related (**Supplementary Figure S2**). Briefly, 18 tryptophan-related SNPs were identified, explaining 3.80% of the circulating tryptophan levels’ variance and 4 kynurenine-related SNPs were selected, explaining 1.19% of circulating kynurenine levels’ variance. The strongest signal identified in association to tryptophan was rs13122250 (P = 8.95×10^‐12^) on chromosome 4, which has not been investigated yet. The second strongest signal was rs1016522 (P = 1.59×10^‐10^), identified within the HMHB1 gene, which is responsible for the generation of immune response after recognition by specific T cells (50), and involved in adaptive immune response, cellular response to tumor necrosis factor and positive regulation of interferon‐gamma production (51). We have identified other genomic loci with less obvious linkage to tryptophan, such as TGFBR3 (rs284191, P = 1.97×10^‐9^), ERGIC1 (rs1559063, P = 7.82×10^‐9^), DGKB (rs38271, P = 1.19×10^‐8^), FTO (rs2111118, P = 1.21×10^‐8^), ZPR1 (rs603446, P = 1.38×10^‐8^), EDIL3 (rs1373962, P = 2.71×10^‐8^), P3H2 (rs710580, P = 3.57×10^‐8^), GREB1 (rs7584842, P = 4.15×10^‐8^). TGFBR3 is involved in immune response (52), while FTO (53) and P3H2 (54) influence tumor occurrence and development. As for kynurenine, the strongest signal identified was rs8051149 within the SLC7A5 gene (P = 9.07×10^‐26^), which is responsible for L‐tryptophan transmembrane transport (55), the positive regulation of cytokine production in immune response, and the positive regulation of interferon‐gamma production (56). Other associations with no obvious link to kynurenine include SH2B3 (rs3184504, P = 6.05×10^‐18^), which is also associated with tumor occurrence and development (57) and IDO2 (rs10085935, P = 3.33×10^‐9^). IDO2 catalyzes the first rate limiting step of the tryptophan catabolism and kynurenine pathway (58) and is involved in immune regulation, however, it may not have a significant role in tryptophan‐related tumoral resistance (59). These SNPs regarded here as instrumental variables, have been verified by previous studies (60–63) (**Supplementary Table S6**). Detailed characteristics of the SNPs strongly associated with site-specific cancers were summarized in **Supplementary Table S2**. Detailed information of the metabolites (tryptophan and kynurenine) and population data included in the bidirectional MR analysis were described in **Supplementary Tables S3–S5**.

The baseline characteristics by quintiles of dietary tryptophan intake for participants were described in **Supplementary Table S7** separately. Participants with lower dietary tryptophan intake level were more likely to be older, have less years in education, be poorer, have higher C-reactive protein level, have lower healthy eating index score, be females, be non-Hispanic whites, smoke less, drink less, have less regular exercise, be hypertensive patients, have cancers and higher cancer mortality.

### 3.2 Results of Bidirectional MR Analysis of Circulating Tryptophan Levels and Site-Specific Cancers

**Figure 2:**
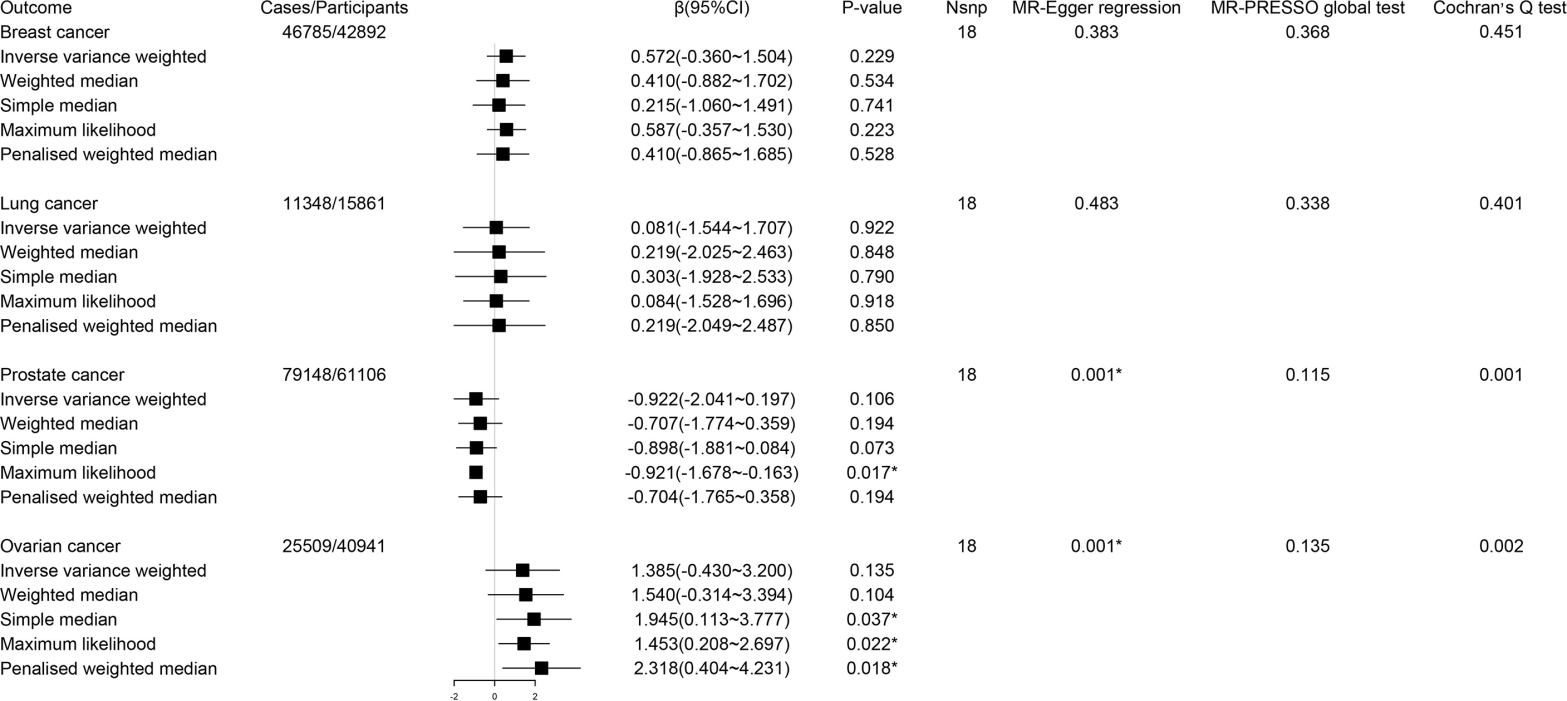
Forest plot for associations between circulating tryptophan levels and the risk of site-specific cancers. β, beta value; CI, confidence interval; Nsnp, number of the SNPs; *P < 0.05.

In MR analysis, genetic predisposition to a lower circulating tryptophan level was not significantly associated with the risk of breast cancer (β 0.57; 95% CI -0.36-1.50, *P* = 0.23), lung cancer (β 0.08; 95% CI -1.54-1.71, *P* = 0.92), prostate cancer (β -0.92; 95% CI -2.04-0.20, *P* = 0.11) and ovarian cancer (β 1.39; 95% CI -0.43-3.20, *P* = 0.14) (**Figure 2**). Since significant heterogeneity for the associations of circulating tryptophan levels with prostate cancer and ovarian cancer were obtained by Cochran’s Q test, a multiplicative random effects model (inverse variance weighted regression) was adopted to re-estimate causal effects again (prostate cancer, *P* = 0.11; ovarian cancer, *P* = 0.14), which indicated similar results. Because MR-Egger regression showed significant horizontal pleiotropy for the associations of circulating tryptophan levels with prostate cancer and ovarian cancer, the results of MR Egger based on sensitivity analysis were used to estimate the causal relationships (prostate cancer: β 1.18; 95% CI -14.19-16.55, *P* = 0.88) (ovarian cancer: β -2.36; 95% CI -27.48-22.77, *P* = 0.88), which were consistent with the results of the IVW method (30).

**Figure 3:**
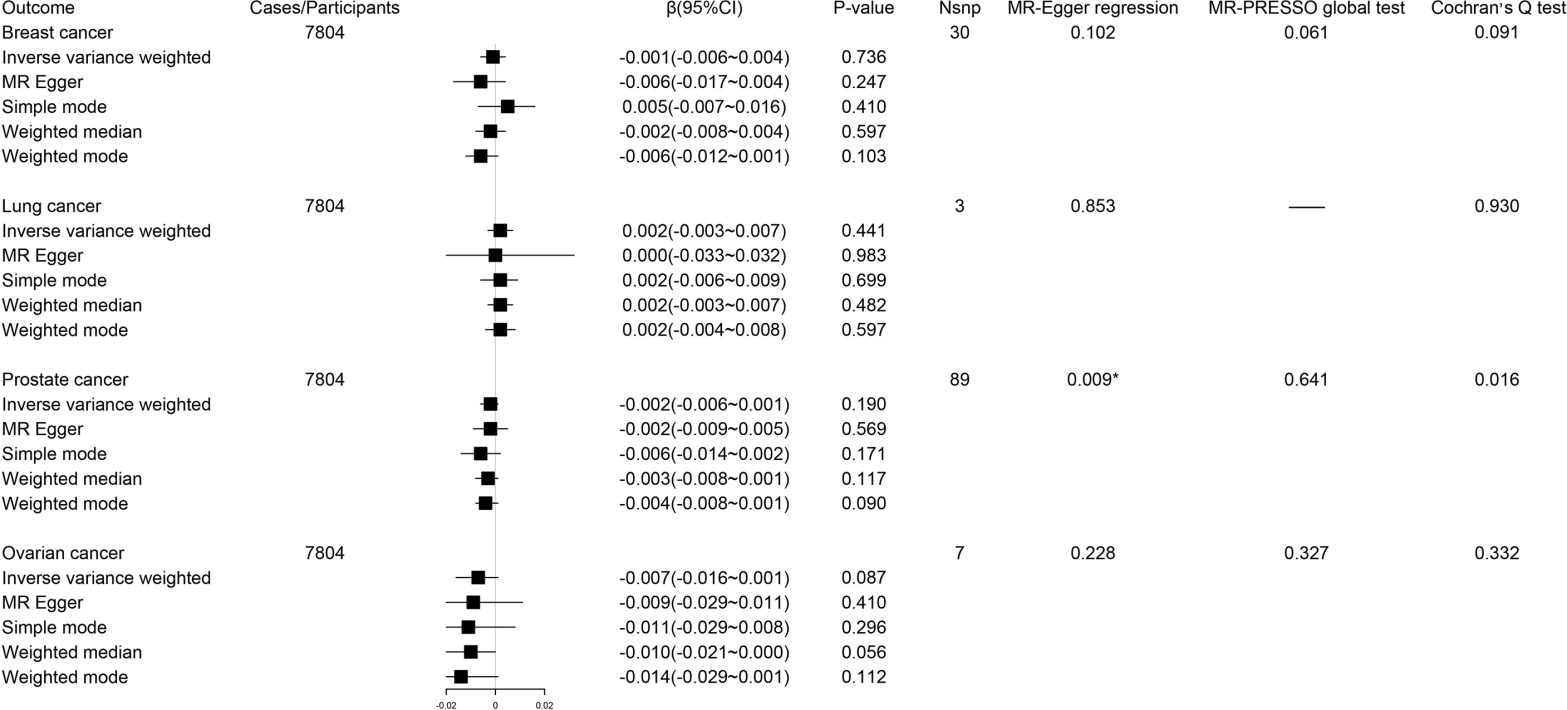
Forest plot for associations between site-specific cancers and circulating tryptophan levels. β, beta value; CI, confidence interval; Nsnp, number of the SNPs; *P < 0.05.

During reverse MR analysis, the causal effects of site-specific cancers on circulating tryptophan levels were generally not significant (**Figure 3**). Due to the existence of horizontal pleiotropic and heterogeneity suggested by MR-Egger regression and Cochran’s Q test, the weighted median method was conducted to verify the association of prostate cancer with circulating tryptophan levels, which displayed consistent results compared with IVW approach (β -0.003; 95% CI -0.008-0.001, *P* = 0.12). The forest plots and scatter plots for the causal effects of exposures on corresponding outcomes were exhibited in **Supplementary Figures S3–S10**.

### 3.3 Results of Bidirectional MR Analysis of Circulating Kynurenine Levels and Site-Specific Cancers

**Figure 4:**
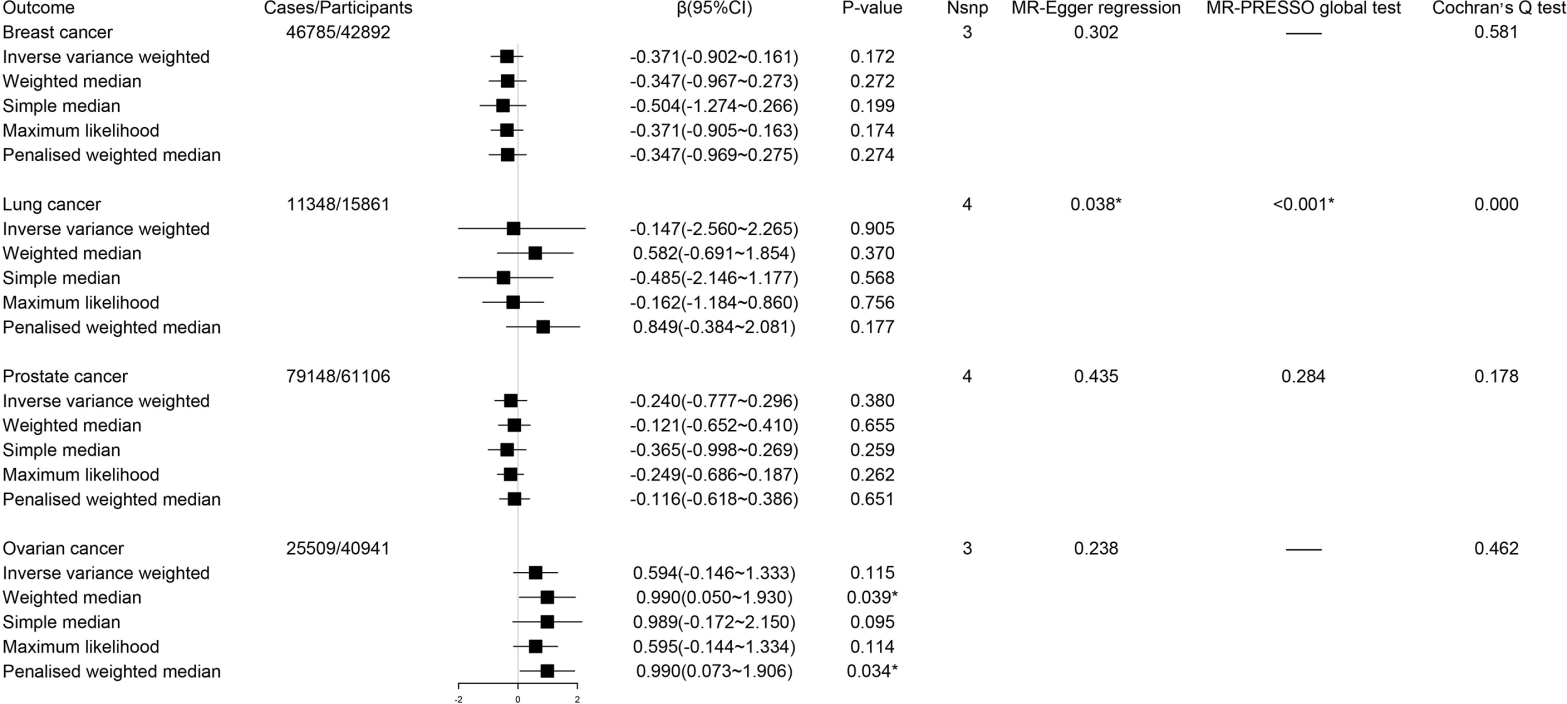
Forest plot for associations between circulating kynurenine levels and the risk of site-specific cancers. β, beta value; CI, confidence interval; Nsnp, number of the SNPs; *P < 0.05.

Using IVW method as our primary method, we found the estimates of the causal relationship between circulating kynurenine levels and the risk of breast cancer (β -0.37; 95% CI -0.90-0.16, *P* = 0.17), lung cancer (β -0.15; 95% CI -2.56-2.27, *P* = 0.91), prostate cancer (β -0.24; 95% CI -0.78-0.30, *P* = 0.38) and ovarian cancer (β 0.59; 95% CI -0.15-1.33, *P* = 0.12) were not significant in MR analysis (**Figure 4**). Just as described before, inverse variance weighted regression should be regarded as a main method during MR analysis of circulating kynurenine levels with lung cancer (ovarian cancer, *P* = 0.91), which suggested no significant associations of circulating kynurenine levels with lung cancer.

**Figure 5:**
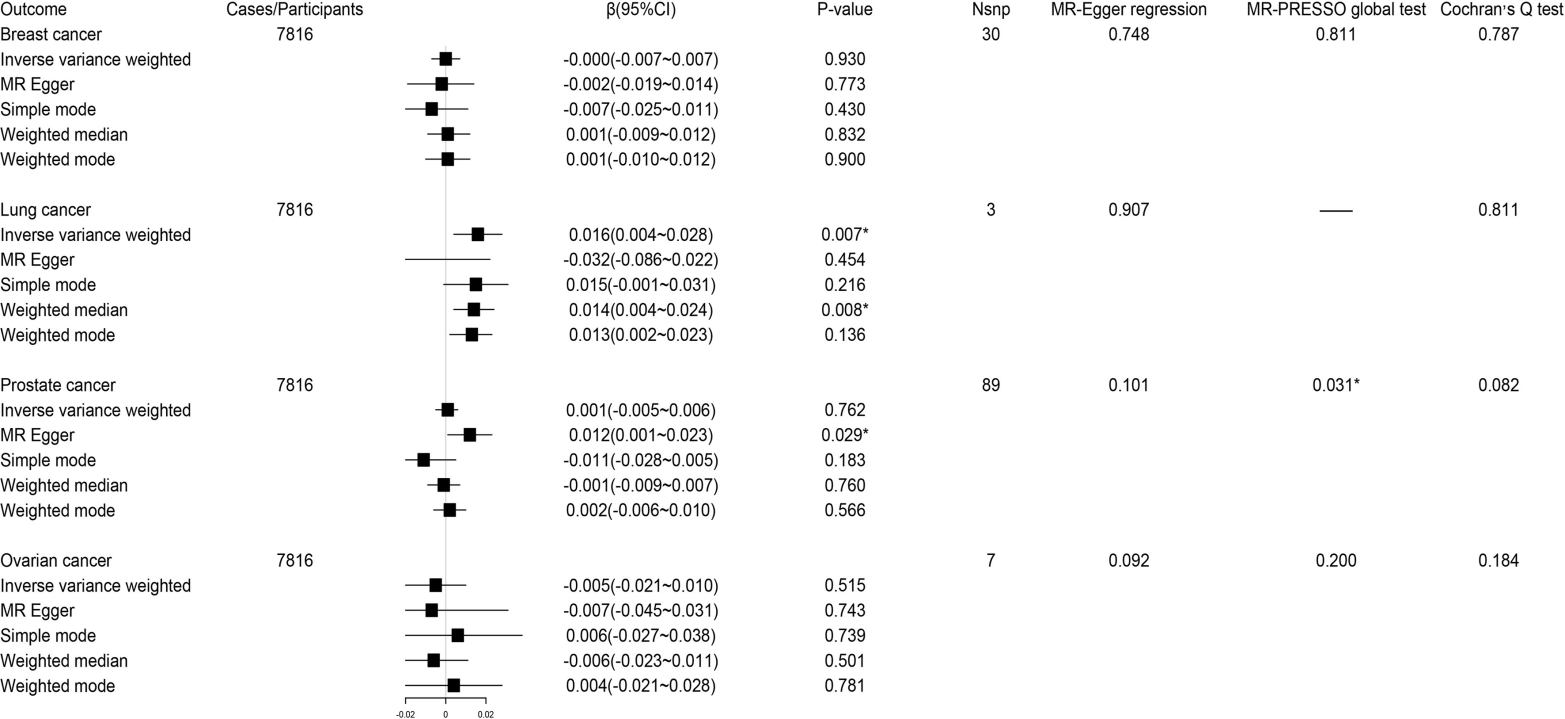
Forest plot for associations between site-specific cancers and circulating kynurenine levels. β, beta value; CI, confidence interval; Nsnp, number of the SNPs; *P < 0.05.

The reverse MR analysis showed lung cancer, but not other cancers, had significant genetic correlation with circulating kynurenine levels (**Figure 5**). Considering there were only three instrumental variables for lung cancer explaining 0.39% of the variance of the risk of lung cancer, the precision of this estimate may be relatively limited. Since the *P*-value of MR-PRESSO global test on the reverse MR analysis of prostate cancer and circulating kynurenine levels reached the significant level, MR Egger based on sensitivity analysis was used to estimate causal effects again (β 0.012; 95% CI 0.001-0.023, *P* = 0.03), which indicated there might be a weak correlation between prostate cancer and circulating kynurenine levels. The forest plots and scatter plots of causal effects from exposures to corresponding outcomes were shown in **Supplementary Figures S11–S18**.

### 3.4 Observational Study of Dietary Tryptophan Intake With All-Cancer Mortality

**Supplementary Table S8** presents the association between dietary tryptophan intake and all-cancer mortality. During the analysis of the overall sample, model 1 adjusting for age, gender and race did not show any association in quintile 2 (HR 1.004; 95% CI 0.739-1.365), quintile 3 (HR 0.997; 95% CI 0.703-1.413), quintile 4 (HR 1.137; 95% CI 0.825-1.567) and quintile 5 (HR 1.154; 95% CI 0.834-1.597) (quintile 1 was considered the reference group, *P*_trend_ = 0.75).With adjustments for age, gender, race, education, poverty income ratio, smoker, drinker and regular exercise in model 2, HRs (95% CIs) in quintile 2 (HR 1.066; 95% CI 0.788-1.442), quintile 3 (HR 0.994; 95% CI 0.703-1.413), quintile 4 (HR 1.163; 95% CI 0.856-1.581) and quintile 5 (HR 1.233; 95% CI 0.942-1.612) suggested no statistically significant (*P*_trend_ = 0.57). After further adjusting for healthy eating index score, C-reactive protein and diabetes in model 3, HRs (95% CIs) for all-cancer mortality were 1.151 (0.847-1.563) in quintile 2, 1.078 (0.757-1.533) in quintile 3, 1.205 (0.881-1.648) in quintile 4 and 1.160 (0.872-1.545) in quintile 5, which still remained not significant (*P*_trend_ = 0.80). Furthermore, the results of the COX regression model analysis conducted in cancer and non-cancer population were consistent with that of the whole study population. Generally speaking, hazard ratios and 95% CIs for all-cancer mortality by quintiles of dietary tryptophan intake did not show any significant effects.

## 4 Discussion

In this study, the results of the MR analysis following sensitivity analysis, did not provide any evidence to prove the causal relationship between tryptophan and kynurenine and the risk of site-specific cancers. Moreover, after statistical analysis of dietary tryptophan intake and all-cancer mortality in NHANES III, we did not find any possible connection.

Cancer, which is characterized by uncontrolled growth and metastasis, is a general term for a group of multiple diseases that can affect any part of the body, and it is the second leading cause of death worldwide. The occurrence and development of cancer is closely related to the immune system. The theory of cancer immune monitoring proves the interaction between cancer and the immune system (64). A large amount of evidence shows that both the innate and the acquired immune response can identify and eliminate tumors and mutated cancer cells are easily recognized and eliminated by the human immune system.

In recent years, more and more factors thought to have anti-tumor immunosuppressive effects have been discovered. The promoting effect of the reduction of tryptophan on the risk of cancer has been found in some observational studies, which basically involves the most types of cancer.

Two points of view have been proposed to explain how reduction of tryptophan plays an important role in immune process. One view is that the reduction of tryptophan inhibits the proliferation of immune cells by significantly reducing the content of tryptophan (12, 65, 66). Another point of view supports that it is not tryptophan itself which is responsible for this effect, but rather the changes of some metabolites involved in the tryptophan catabolism pathways that play a leading role in immunosuppression (67, 68). A generally accepted view is that tryptophan can be catabolized by immune cells and cancer cells at sites of immunodominance, inflammation, and tumorigenesis.

The body suppresses the production of antigen-specific T cells and limits excessive immune responses by depleting tryptophan and accumulating tryptophan catabolites with immunosuppressive effects. For example, after observing the chronic inflammation C57BL/6 strain in mice for a week, which was produced by threating them with phorbol myristate acetate, researchers found that the decrease in tryptophan was related to colon cancer and other inflammation-driven cancers (17). After measuring and analyzing the levels of tryptophan metabolites in 80 patients with colorectal cancer, it was found that the decrease in plasma tryptophan concentrations was associated with more advanced cancers (69). Another cross-sectional study of 200 patients with T cells leukemia/lymphoma designed to screen for immune activation-related biomarkers showed that lower concentration of plasma tryptophan was associated with shorter survival time in cancer patients (21). In addition, the viewpoint that the common phenomenon of decreased tryptophan and increased kynurenine concentrations in peripheral blood predicting enhanced tryptophan catabolism in cancer patients is related to the activation of pro-inflammatory cytokines, tumor progression and the occurrence of adverse clinical outcomes is proposed in more and more researches (12).

However, after excluding confounding factors such as socio-economic, diet and lifestyle, our study did not find any clear causal association between circulating tryptophan levels and the risk of site-specific cancers. We speculate the reduction in concentrations of tryptophan may not have enough effects on the immune response to tumors to change the immune system’s role in the development of cancer. Alternatively other substances of the tryptophan degradation pathway affect the occurrence and development of cancer, rather than the decrease of tryptophan itself.

This is also consistent with other previous studies, in which the association between tryptophan concentrations and the occurrence and development of cancer was not significant. Studies on the changes in tryptophan metabolism pathways during pregnancy and infection found that tryptophan metabolites were the key regulators that regulate the behavior of immune cell behavior, and decrease in tryptophan was only an accompanying phenomenon indicating the changes in pathways (8). Although in most *in vitro* experiments, increasing the concentration of tryptophan in culture medium can restore the growth of cancer cells, bacteria or parasites, there are still many potential reasons that can be used to suspect this view. One reason is that tryptophan depletion experiments performed under cell culture conditions can not fully represent the internal environment of the body associated with infection. Another is that most bacteria can synthesize the required tryptophan by themselves, which means the effect of local tryptophan reduction may be amplified.

In the study of the relationship between dietary tryptophan intake and the risk of cancer death in NHANES III and in consistency with the MR analysis based on the whole study population and the cancer-affected population, we came to the conclusion that dietary tryptophan intake has little effect on the occurrence and development of cancer death after adjusting for age, gender, race, education, poverty income ratio, smoking, drinking, regular exercise, healthy eating index score, serum C-reactive protein and diabetes. The reason may be that the demand for tryptophan is multifaceted, such as protein synthesis, neuron protection, the maintenance of signal pathways, immune tolerance and the synthesis of nicotinamide adenine dinucleotide etc. Circulating kynurenine is primarily derived from endogenous tryptophan catabolism and current data on the presence and content of kynurenine in food are unexpectedly sparse, and thus it was not possible to conduct an association analysis between dietary kynurenine intake and cancer mortality based on NHANES data.

The decrease in circulating tryptophan concentrations in the human body is believed to be mainly caused by the enhancement of the tryptophan catabolism (3). Generally speaking, in the process of tryptophan catabolism, circulating tryptophan concentrations continues to decrease, and its downstream metabolites are continuously produced at the same time. Based on the above description, we speculate that the regulation of immune response in the tumor microenvironment produced by tryptophan catabolism pathways, is mostly due to the impact of substances of the dominant metabolic pathways, other than tryptophan.

Tryptophan is an important precursor of biologically active metabolites including tryptamine, serotonin, melatonin, kynurenine, kynurenic acid, quinolinic acid and nicotinamide adenine dinucleotide, which are mainly produced through three different metabolic pathways: serotonin pathway, kynurenine pathway and indole pathway (70, 71). Among them, more than 95% of free tryptophan is degraded *via* the kynurenine pathway (72).

More and more evidence from multiple laboratories indicate that the increase of kynurenine and its metabolites with immunomodulatory properties is the main mechanism of promoting immune tolerance in the tryptophan catabolism. Disturbances of the kynurenine pathway are thought to be related to central nervous system diseases, malignant tumors, inflammatory bowel diseases and cardiovascular diseases. Kynurenine can reduce the activity of natural killer cells, dendritic cells and T cells. Kynurenic acid can promote monocyte extravasation and control the release of cytokines (6).

As an endogenous pro-tumor proliferation ligand, kynurenine can bind to aromatic hydrocarbon receptors and activate aromatic hydrocarbon receptors to exert its biological effects, which implies that the high levels of kynurenine may increase the proliferation and migration of cancer cells and help tumors escape from immune surveillance (73). During *in vitro* experiments, exogenous addition of kynurenine, 3-hydroxykynurenine, 3-hydroxyanthranilic acid and quinolinic acid inhibited the proliferation of cultured T cells and induced them to apoptosis (5, 74).

However, our study did not find any evidence on the association of circulating kynurenine levels with the risk of site-specific cancers, which may be due to the slight alteration of kynurenine and its downstream metabolites in the tumor microenvironment. Similar to the effect of decreased tryptophan on anti-tumor immune response in the body, the effect of increased kynurenine on immune system may also be little. During a number of observational studies, although patients with gynecological cancer (13), T cells leukemia (21), colorectal cancer (12), malignant melanoma (11), malignant glioma (3, 10) and lung cancer (9) had reduced systemic tryptophan levels, no increase in the concentration of kynurenine pathway metabolites in the blood was observed.

After summarizing and analyzing all the results of this research, we put forward two guesses: 1) the changes of tryptophan and its main metabolite kynurenine are related to the immune response, but the inhibitory effects of the decrease of tryptophan and the increase of kynurenine on the anti-tumor immune response may not be enough to affect the occurrence and development of cancer; 2) there is a strong causal link between other certain substances in tryptophan metabolism pathways and the risk of cancer. The changes in circulating tryptophan and kynurenine are only accompanying phenomena of the progress of these pathways.

Preclinical studies have shown TDO and IDO, the main rate-limiting enzymes that can regulate kynurenine pathway in patients with malignant tumors, can regulate the tryptophan-mediated tumor immune escape response through depleting tryptophan and accumulating kynurenine in the tumor microenvironment (1). But whether and how the decrease of tryptophan or the increase of kynurenine promotes T cells-mediated tumor rejection *in vivo* remains to be studied. Existing studies provide some possible mechanisms. For example, the reduction of tryptophan leads to anergy and apoptosis of T cells through the general amino acid control non-derepressible 2 (75) (GCN2) and the integrated stress response (76) (IRS) pathways and an increase in kynurenine inhibits T cells differentiation through the aryl hydrocarbon receptor (3) (AHR) pathway. The fact that a variety of human tumors express TDO and IDO indicates the therapeutic potential of targeted drugs to inhibit TDO or IDO in the process of cancer treatment (77–79). However, current clinical trials have shown although some of the key enzyme inhibitors have achieved the expected effects in early cancer immunotherapy, the results of phase III trial are negative, which suggests we lack a precise understanding of the exact downstream mechanism of immunosuppression related to tryptophan metabolism.

Components of the tryptophan catabolism that were previously associated to cancer, have been found to interact with pathways of the tumor microenvironment (80, 81). IDO for example, has been reported to be associated with changes in the complement pathway of the tumor microenvironment (82), while interferons which are potent inducers of immunomodulatory responses are mediated by IDO (83). IDO also regulates the activation of tumor suppressive regulatory T cells in the tumor microenvironment (84). Another important immunosuppressive cell population of the tumor microenvironment, the myeloid‐derived suppressor cells (MDSCs) are recruited to tumors by an IDO-indirect mechanism (85). In conclusion, many physiological processes are capable of inducing IDO, and multiple factors may limit IDO expression and thus regulate IDO activity in physiological environments. Therefore, we should also acknowledge that interactions between genetics and environment may still increase the risk of cancer in association to the tryptophan and kynurenine pathways.

In addition, the important role of serotonin pathway in cancer progression and anti-tumor immune response is being confirmed by more and more researches. Serotonin is an inflammatory mediator (86) related to the proliferation and invasion of various cancer cells (87). Studies on triple-negative breast cancer have shown serotonin promotes the invasion and proliferation of tumor cells through its receptor subtype 5-HT_7_ (88). In certain cancers and gliomas, serotonin has shown to promote tumor growth and survival (89). In animal experiments, serotonin regulates the expression of specific serotonin receptors in cancer cells through a process called serotonylation and up-regulates the expression level of programmed cell death ligand 1 (PD-L1), which is related to the suppression of the immune system (90).

Our study has several strengths. First, to our knowledge, this is the first bidirectional MR analysis on the relationship between circulating tryptophan or kynurenine and site-specific cancers, which strengthens the causal inference through diminishing residual confounding and other biases. Second, we used the summary statistics of large-scale GWASs. Third, to examine the possible associations, NHANES III data and four kinds of site-specific cancers from different data sources were chosen as our outcomes to increase the statistical power to detect weak associations.

There are still several limitations in our study. The major limitation is that only circulating tryptophan and kynurenine has been studied in the present study, however, other metabolites involved in the tryptophan metabolic pathways have not been studied yet. In addition, the size of the populations used to select genetic instruments strongly associated with tryptophan or kynurenine may not be large enough, which may affect the choice of instrumental variables. Third, despite several large-scale genetic consortia we utilized, the variation of site-specific cancers explained by the SNPs was still relatively small, which may limit the statistical power and precision for the MR analysis. Lastly, our results are mainly based on participants of European ancestry and may not be applicable to other ethnic populations. To date, most GWAS performed are primarily conducted on European populations. Although GWAS in Asian and Latin American populations are increasingly being conducted, the population data available generally suffer from insufficient sample sizes and limited geographic distribution of the population. In addition, differential frequencies of genetic variants that exist between populations with different genetic backgrounds can lead to spurious associations between genetic variants and outcomes. Therefore, currently no additional data of diverse ethnic backgrounds can be used in this study. We expect that future GWAS development, will allow this application.

Based on the “common disease‐common variant” hypothesis, GWAS have been extensively conducted to dissect the genetic components of complex diseases and quantitative traits (90). However, the identified disease‐associated common variants can only explain small part of the corresponding disease heritability. Since, the MR analysis approach relies on GWAS, this inevitably leads to missing heritability of lower frequency variants (91). Association studies of less common variants, include adaptive burden tests, variance‐component tests, combined burden and variance‐component tests, combined association in the presence of linkage test, sum of powered score test and exponential combination test (92, 93). We believe these methods can be implemented in the future to explore missing heritability, fill the gaps of the MR analysis approach and augment current findings.

In summary, this MR analysis did not find evidence to support the causal relationship between circulating tryptophan or kynurenine concentration and cancer. Given the existing results, whether changes in tryptophan metabolism pathways may influence the risk of cancer needs further and broader researches such as clarifying the effects of circulating tryptophan or kynurenine on the immune response of the body and carrying out research on the relationship between other metabolites in the tryptophan metabolic pathways and cancer.

## Data Availability Statement

The original contributions presented in the study are included in the article/**Supplementary Material**. Further inquiries can be directed to the corresponding author.

## Ethics Statement

All studies included in this analysis were approved by local review boards and performed in accordance with the Declaration of Helsinki. All participants gave written informed consent to participate in the study.

## Author Contributions

YL is the guarantor of this work and has full access to all the data in the study and takes responsibility for the integrity of the data and the accuracy of the data analysis. YL and RL conceived the study design. XW, YZ and ZW did the statistical analysis. XW, RL, CW and LL repeated and validated the statistical analysis. XW, RL, YZ, LL and LW wrote the manuscript. All authors provided critical insights of the manuscript. All authors contributed to the article and approved the submitted version

## Funding

All authors were supported by funding from National Natural Science Foundation (82030100 to YL).

## Conflict of Interest

The authors declare that the research was conducted in the absence of any commercial or financial relationships that could be construed as a potential conflict of interest.

## Publisher’s Note

All claims expressed in this article are solely those of the authors and do not necessarily represent those of their affiliated organizations, or those of the publisher, the editors and the reviewers. Any product that may be evaluated in this article, or claim that may be made by its manufacturer, is not guaranteed or endorsed by the publisher.
