# Supplementary material for: Analysis of Tryptophan and Its Main Metabolite Kynurenine and the Risk of Multiple Cancers Based on the Bidirectional Mendelian Randomization Analysis
Source: Front Oncol. 2022 Apr 14;12:852718. doi: 10.3389/fonc.2022.852718 (PMC9046840; doi:10.3389/fonc.2022.852718)
Supplement: Supplementary file 1 [file DataSheet_1.docx]

***Supplementary Material***

**1 Supplementary Figures**

**
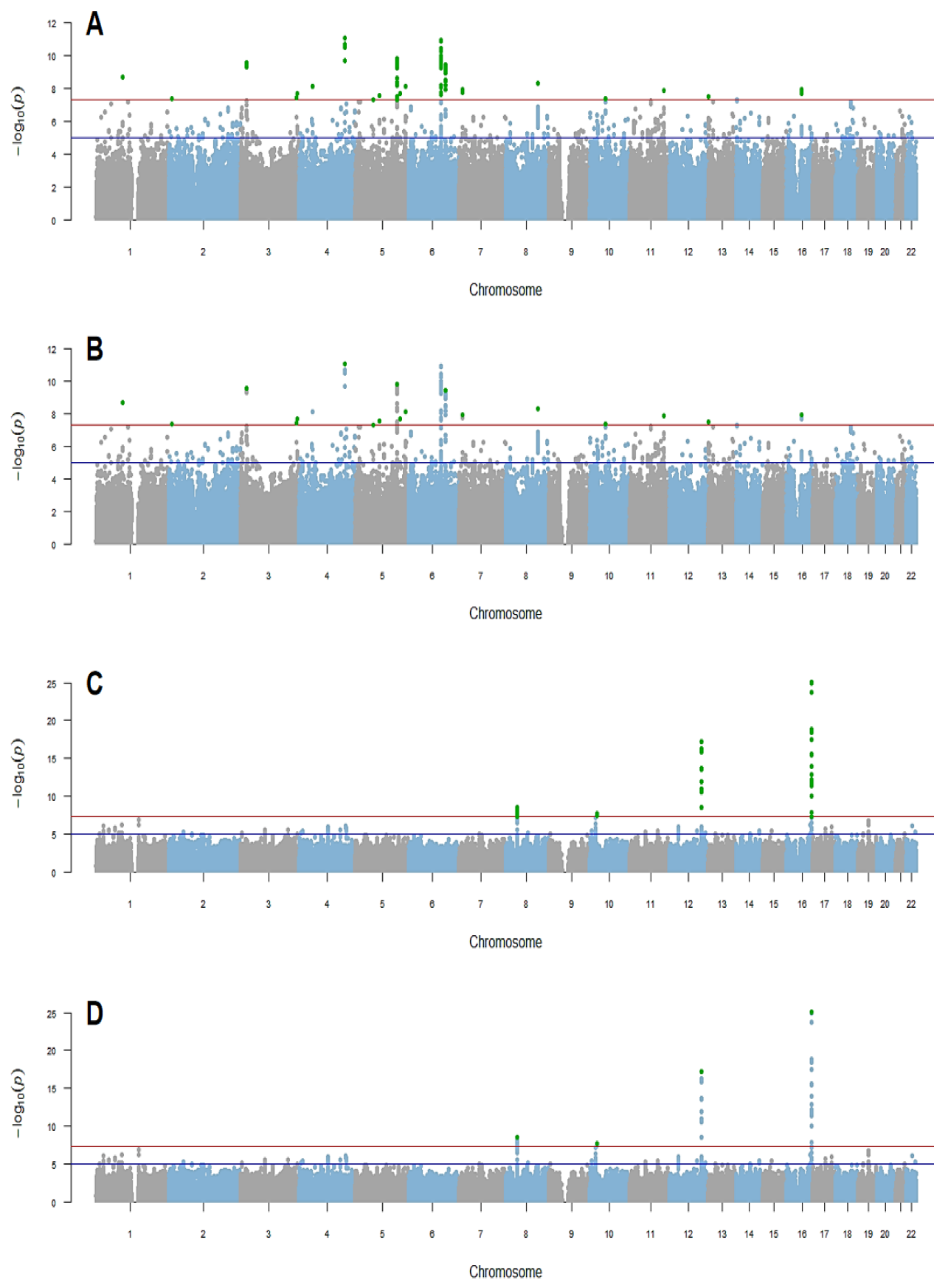
**

**Figure S1.** Manhattan plots of the SNPs strongly associated (*P* < 5 × 10^-8^) with circulating tryptophan/kynurenine levels. A. Filter results of the SNPs strongly associated with circulating tryptophan levels before pairwise-linkage disequilibrium clumping. B. Filter results of the SNPs strongly associated with circulating tryptophan levels after pairwise-linkage disequilibrium clumping. C. Filter results of the SNPs strongly associated with circulating kynurenine levels before pairwise-linkage disequilibrium clumping. D. Filter results of the SNPs strongly associated with circulating kynurenine levels after pairwise-linkage disequilibrium clumping. Each point in the figures represents a SNP. The red solid line is at a *P* value of 5 × 10^-8^. The blue solid line is at a *P* value of 1 × 10^-5^.

**
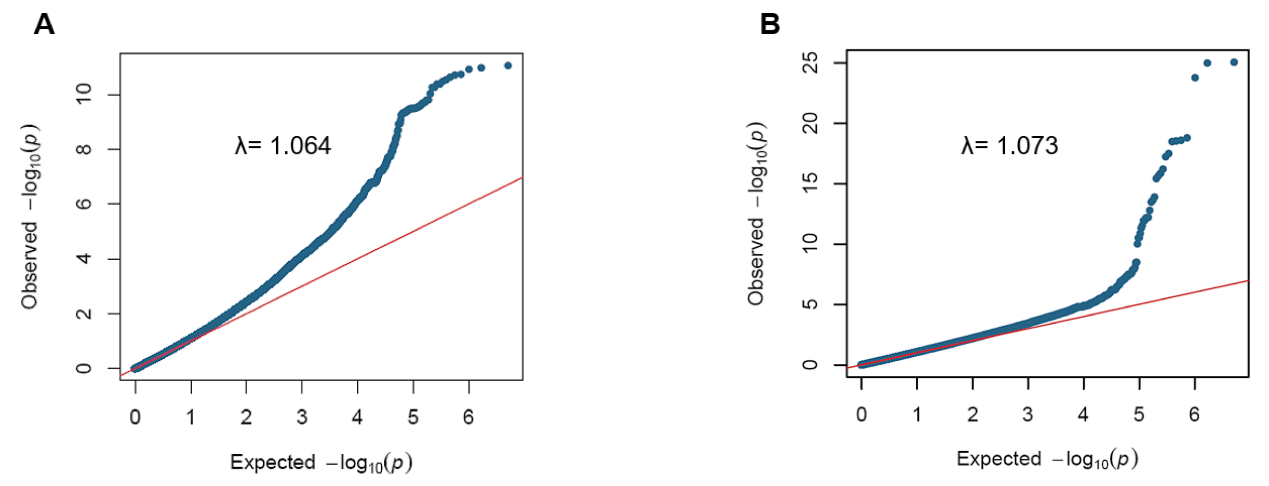
**

**Figure S2.** Quantile-quantile (Q-Q) plots for the genome-wide meta-analysis results of the SNPs strongly associated with circulating tryptophan (A)/kynurenine (B) levels. Each point in the figures represents a SNP.


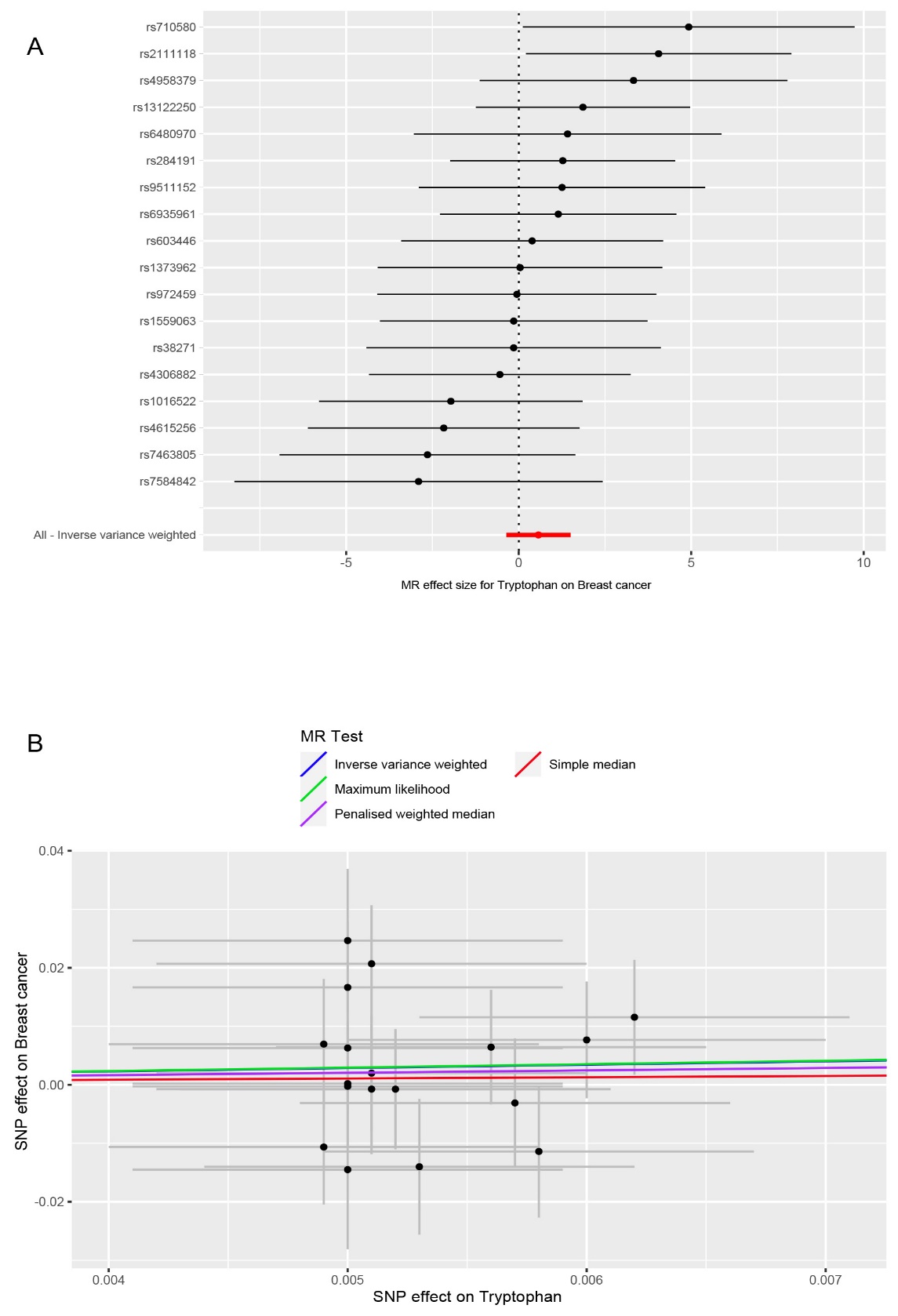


**Figure S3.** Genetically predicted associations between circulating tryptophan levels and the risk of breast cancer. A. Forest plot for the causal effects of circulating tryptophan levels on the risk of breast cancer. B. Scatter plot of different analysis methods for the causal associations between circulating tryptophan levels and the risk of breast cancer. Each approach has a different line. The slope of each line shows the causal association.


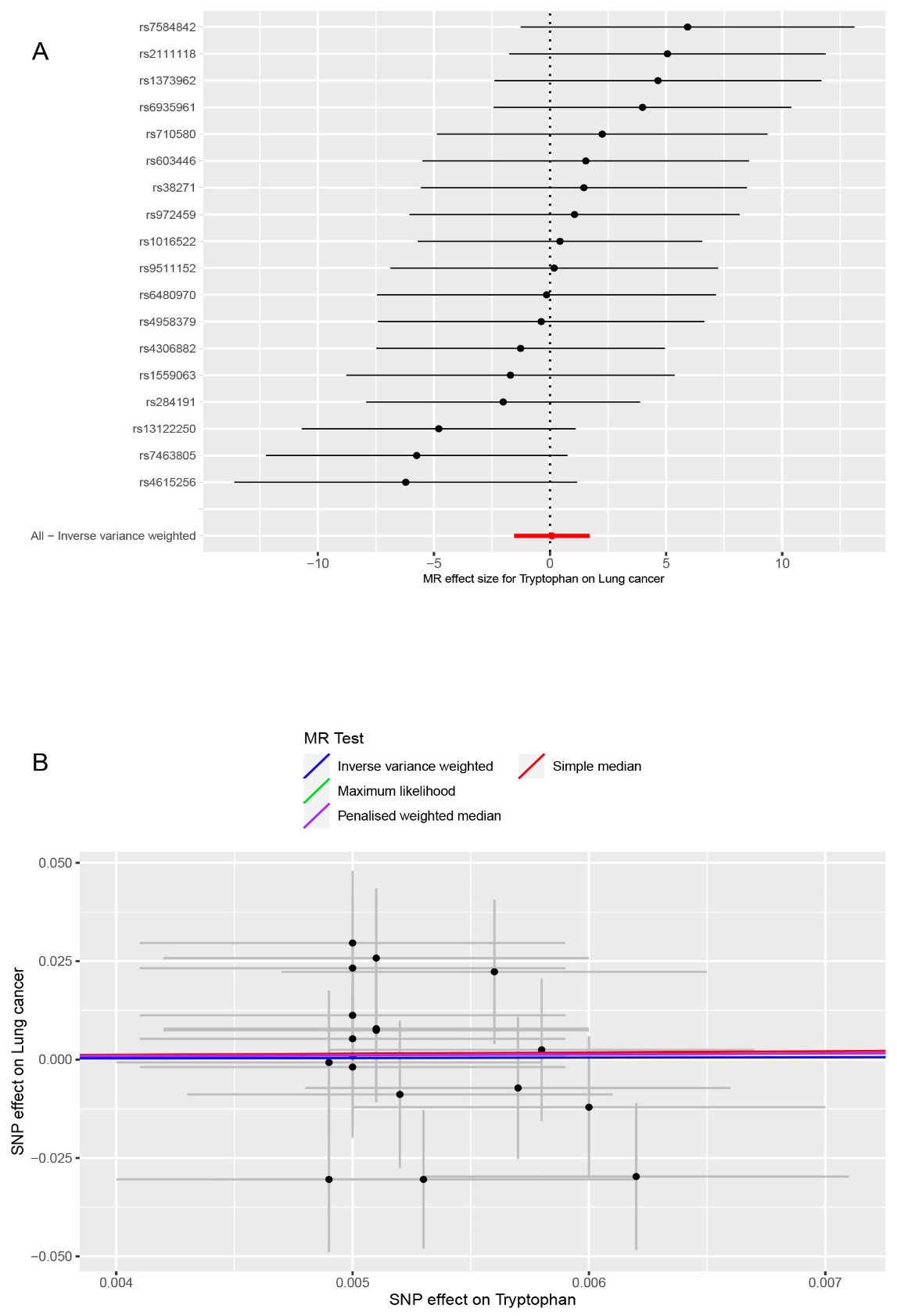


**Figure S4.** Genetically predicted associations between circulating tryptophan levels and the risk of lung cancer. A. Forest plot for the causal effects of circulating tryptophan levels on the risk of lung cancer. B. Scatter plot of different analysis methods for the causal associations between circulating tryptophan levels and the risk of lung cancer. Each approach has a different line. The slope of each line represents the causal association.


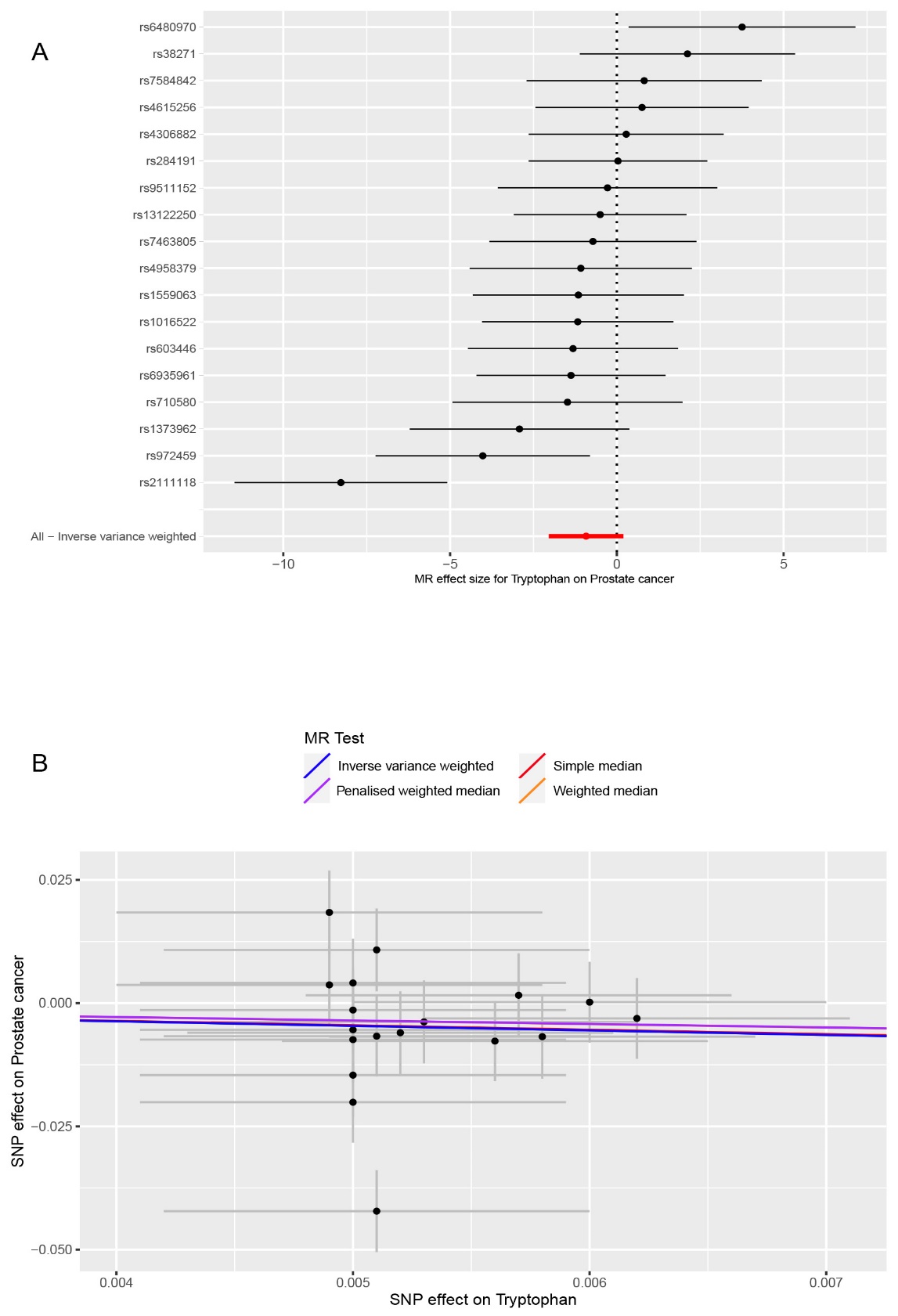


**Figure S5.** Genetically predicted associations between circulating tryptophan levels and the risk of prostate cancer. A. Forest plot for the causal effects of circulating tryptophan levels on the risk of prostate cancer. B. Scatter plot of different analysis methods for the causal associations between circulating tryptophan levels and the risk of prostate cancer. Each approach has a different line. The slope of each line represents the causal association.


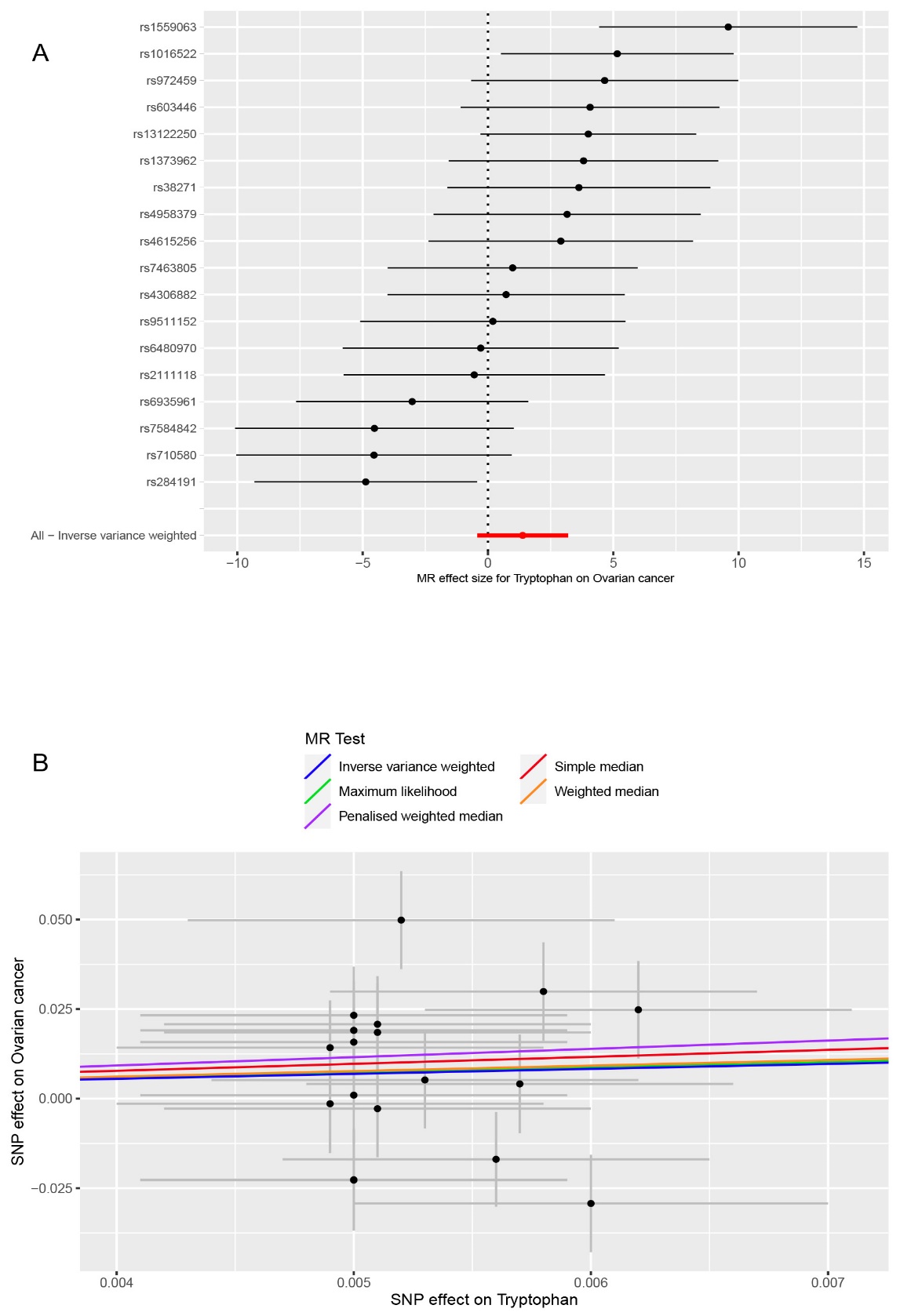


**Figure S6.** Genetically predicted associations between circulating tryptophan levels and the risk of ovarian cancer. A. Forest plot for the causal effects of circulating tryptophan levels on the risk of ovarian cancer. B. Scatter plot of different analysis methods for the causal associations between circulating tryptophan levels and the risk of ovarian cancer. Each approach has a different line. The slope of each line represents the causal association.


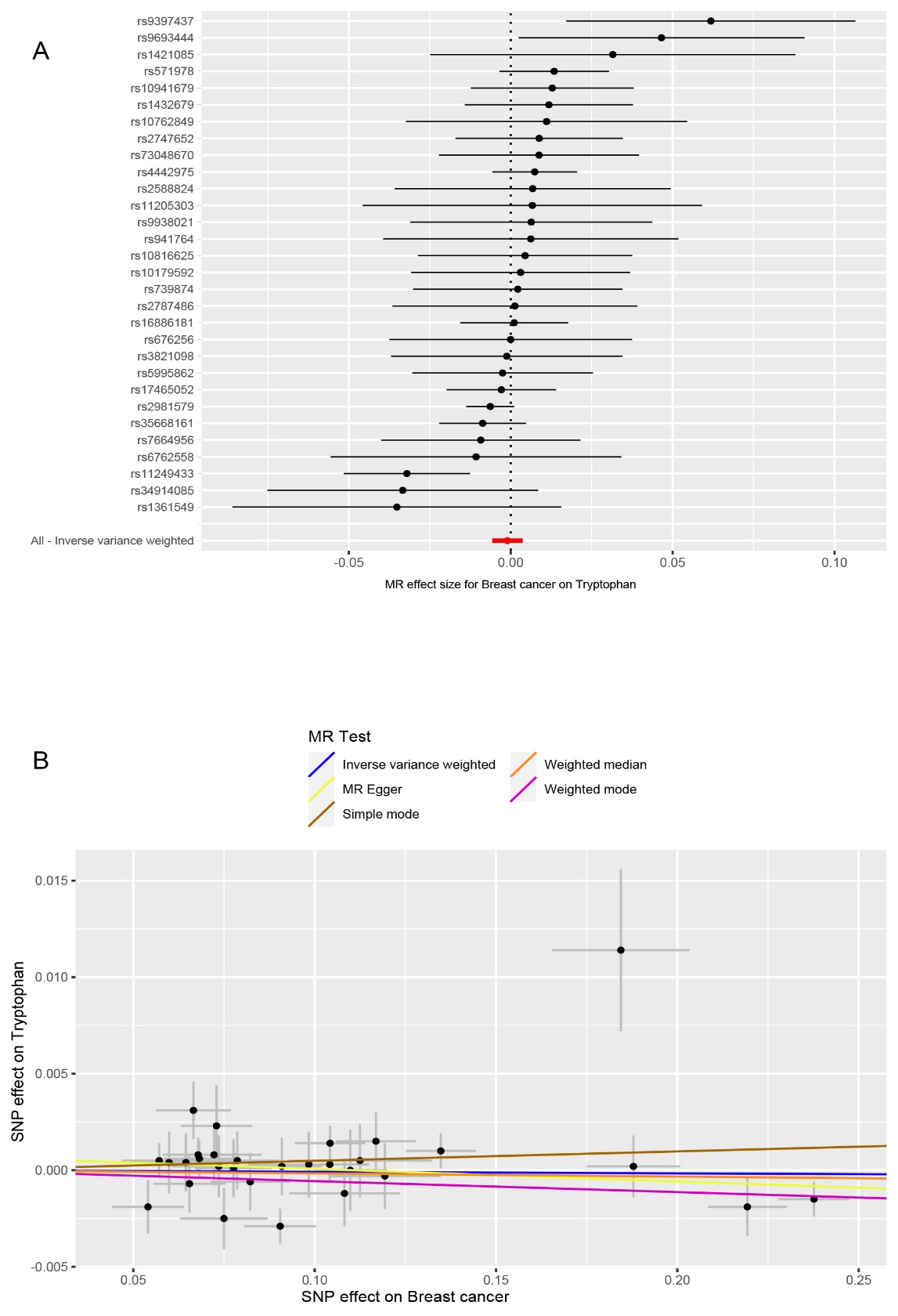


**Figure S7.** Genetically predicted associations between breast cancer and circulating tryptophan levels. A. Forest plot for the causal effects of breast cancer on circulating tryptophan levels. B. Scatter plot of different analysis methods for the causal associations between breast cancer and circulating tryptophan levels. Each approach has a different line. The slope of each line represents the causal association.


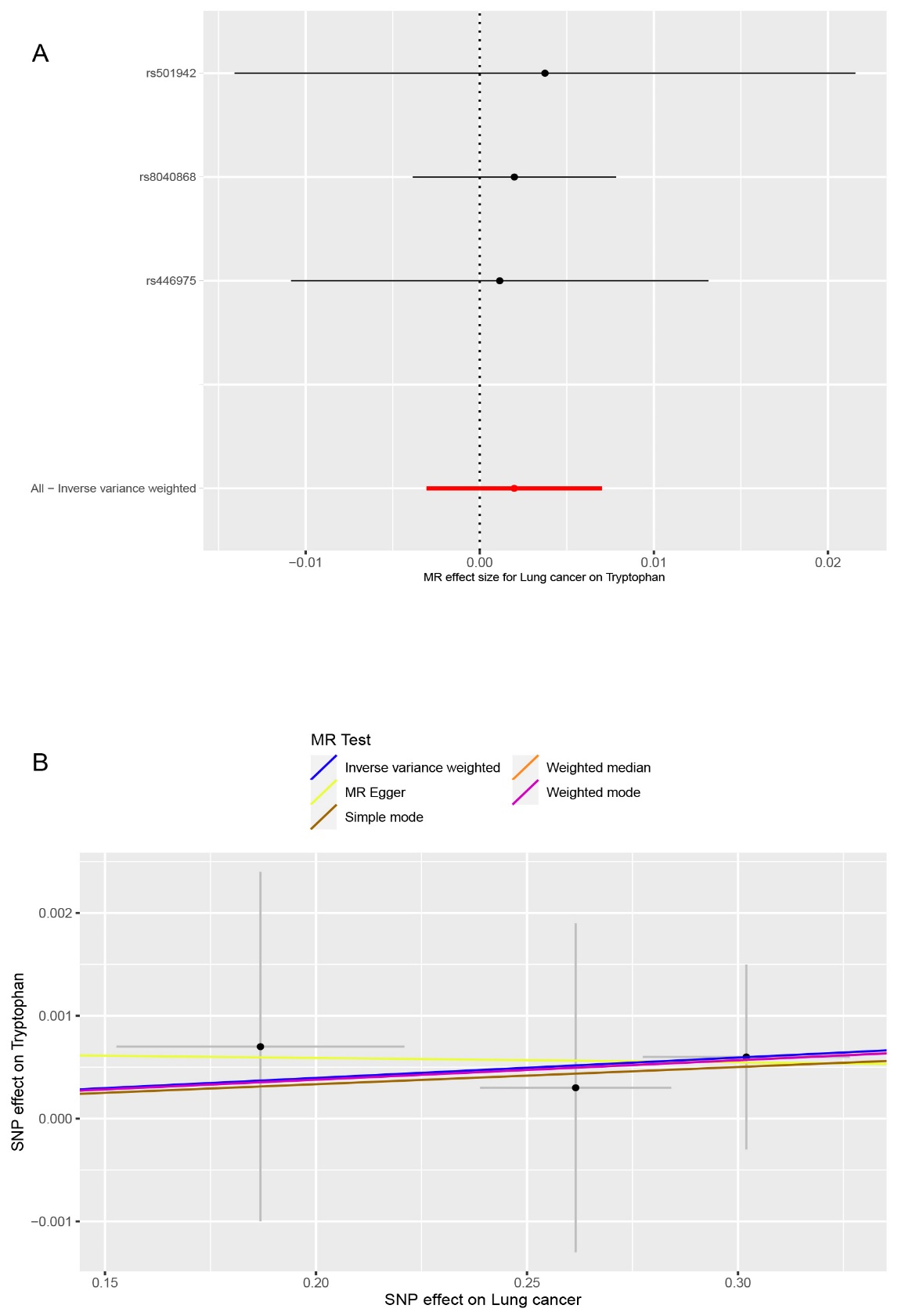


**Figure S8.** Genetically predicted associations between lung cancer and circulating tryptophan levels. A. Forest plot for the causal effects of lung cancer on circulating tryptophan levels. B. Scatter plot of different analysis methods for the causal associations between lung cancer and circulating tryptophan levels. Each approach has a different line. The slope of each line represents the causal association.


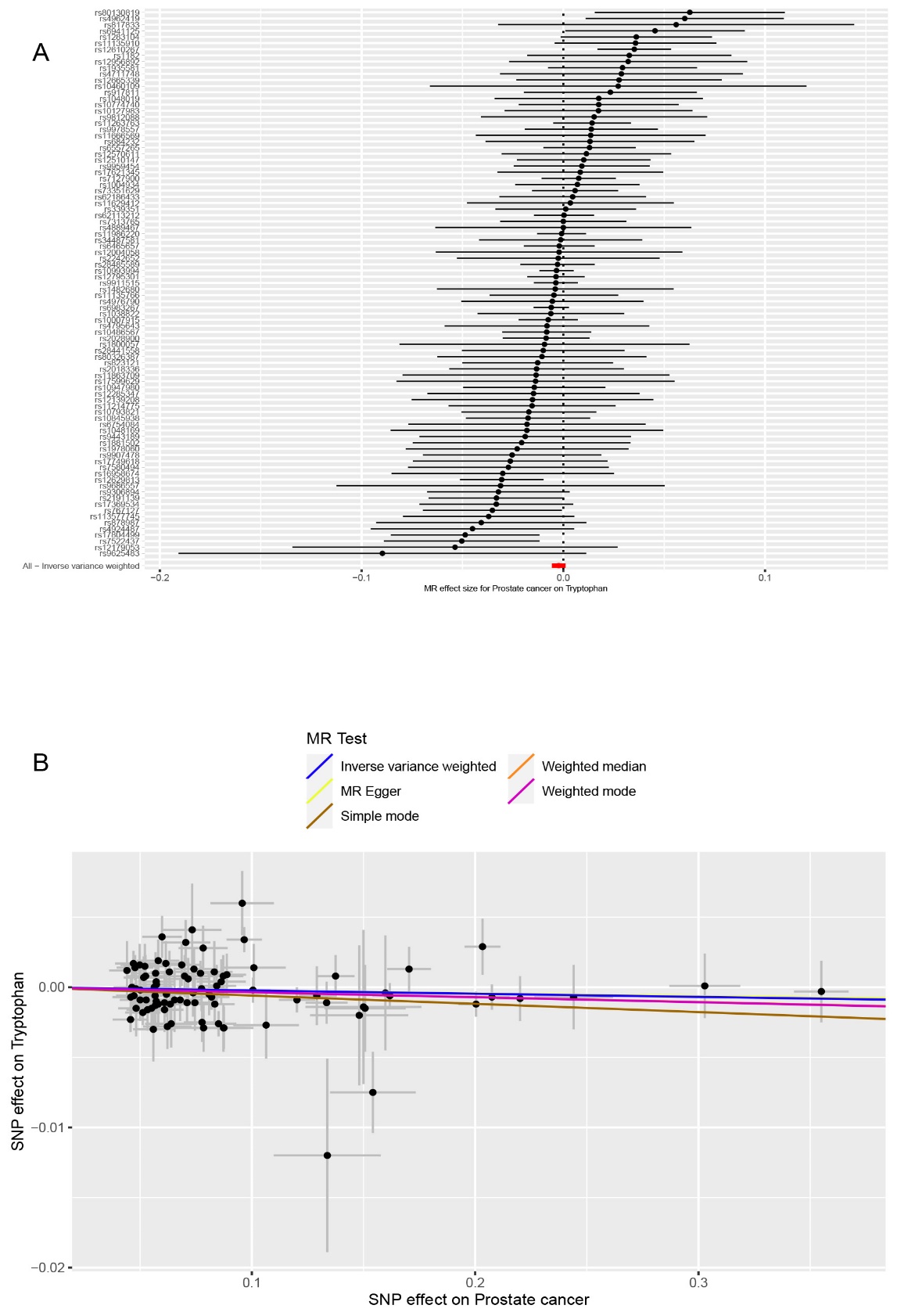


**Figure S9.** Genetically predicted associations between prostate cancer and circulating tryptophan levels. A. Forest plot for the causal effects of prostate cancer on circulating tryptophan levels. B. Scatter plot of different analysis methods for the causal associations between prostate cancer and circulating tryptophan levels. Each approach has a different line. The slope of each line represents the causal association.


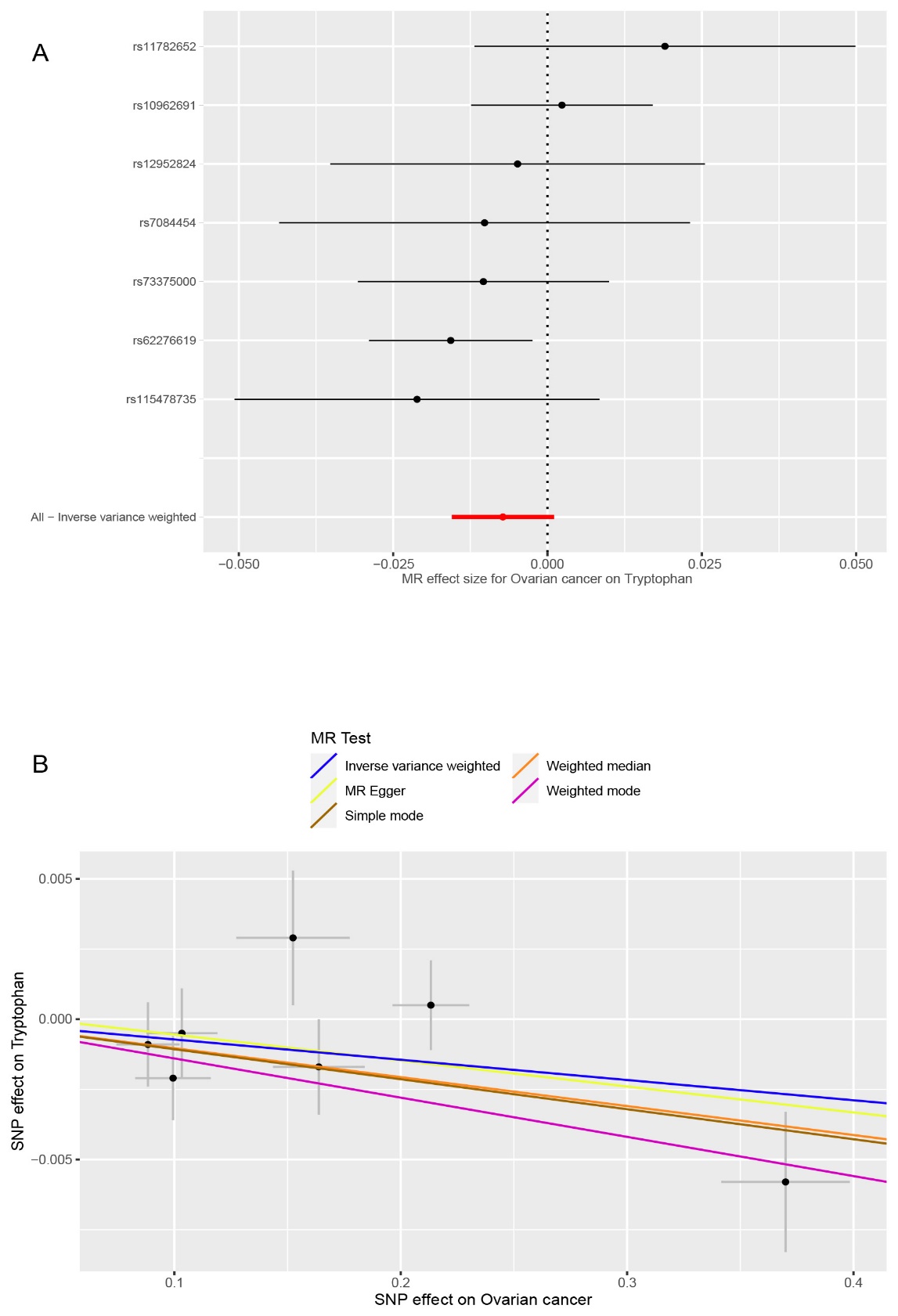


**Figure S10.** Genetically predicted associations between ovarian cancer and circulating tryptophan levels. A. Forest plot for the causal effects of ovarian cancer on circulating tryptophan levels. B. Scatter plot of different analysis methods for the causal associations between ovarian cancer and circulating tryptophan levels. Each approach has a different line. The slope of each line represents the causal association.


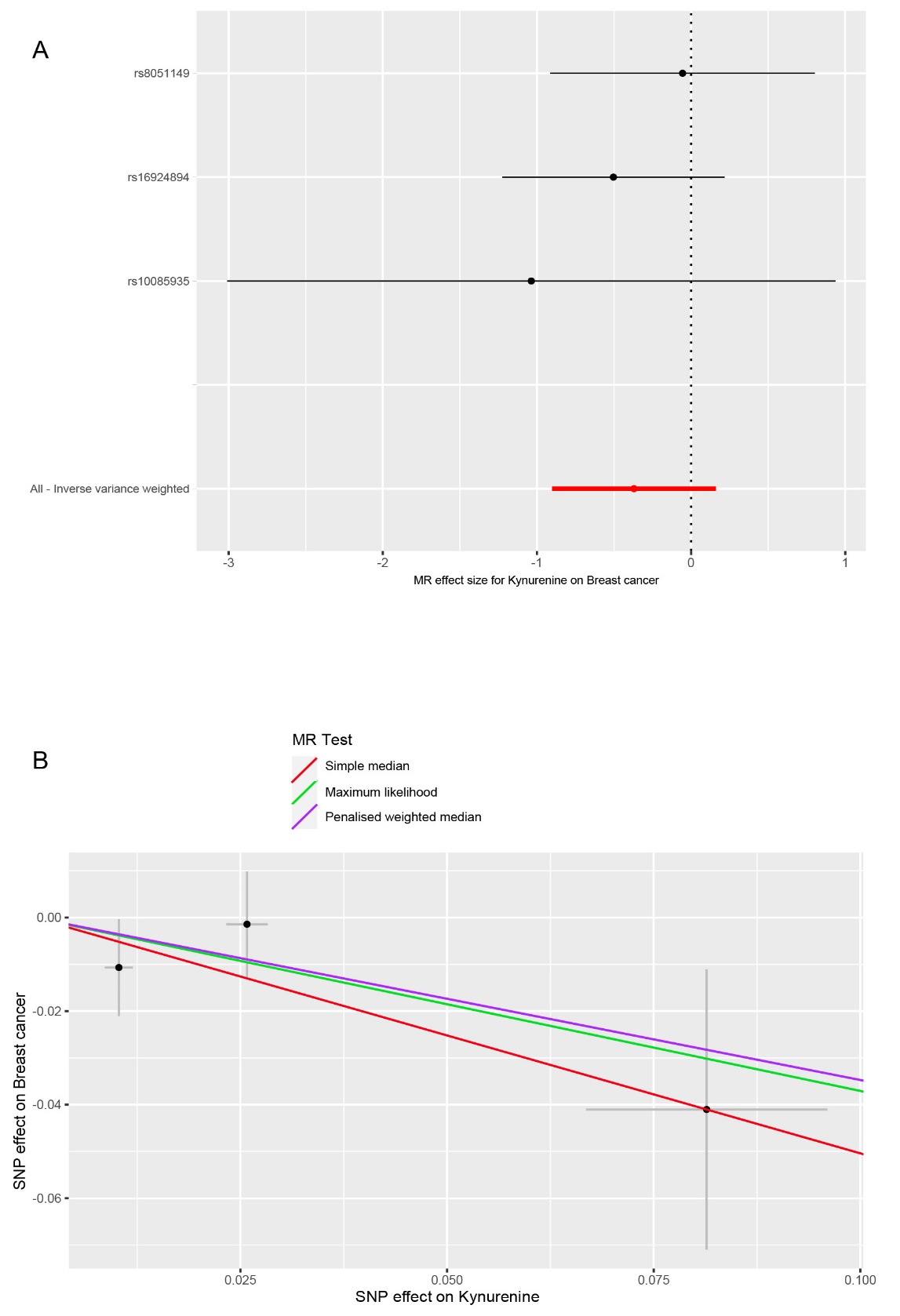


**Figure S11.** Genetically predicted associations between circulating kynurenine levels and the risk of breast cancer. A. Forest plot for the causal effects of circulating kynurenine levels on the risk of breast cancer. B. Scatter plot of different analysis methods for the causal associations between circulating kynurenine levels and the risk of breast cancer. Each approach has a different line. The slope of each line represents the causal association.


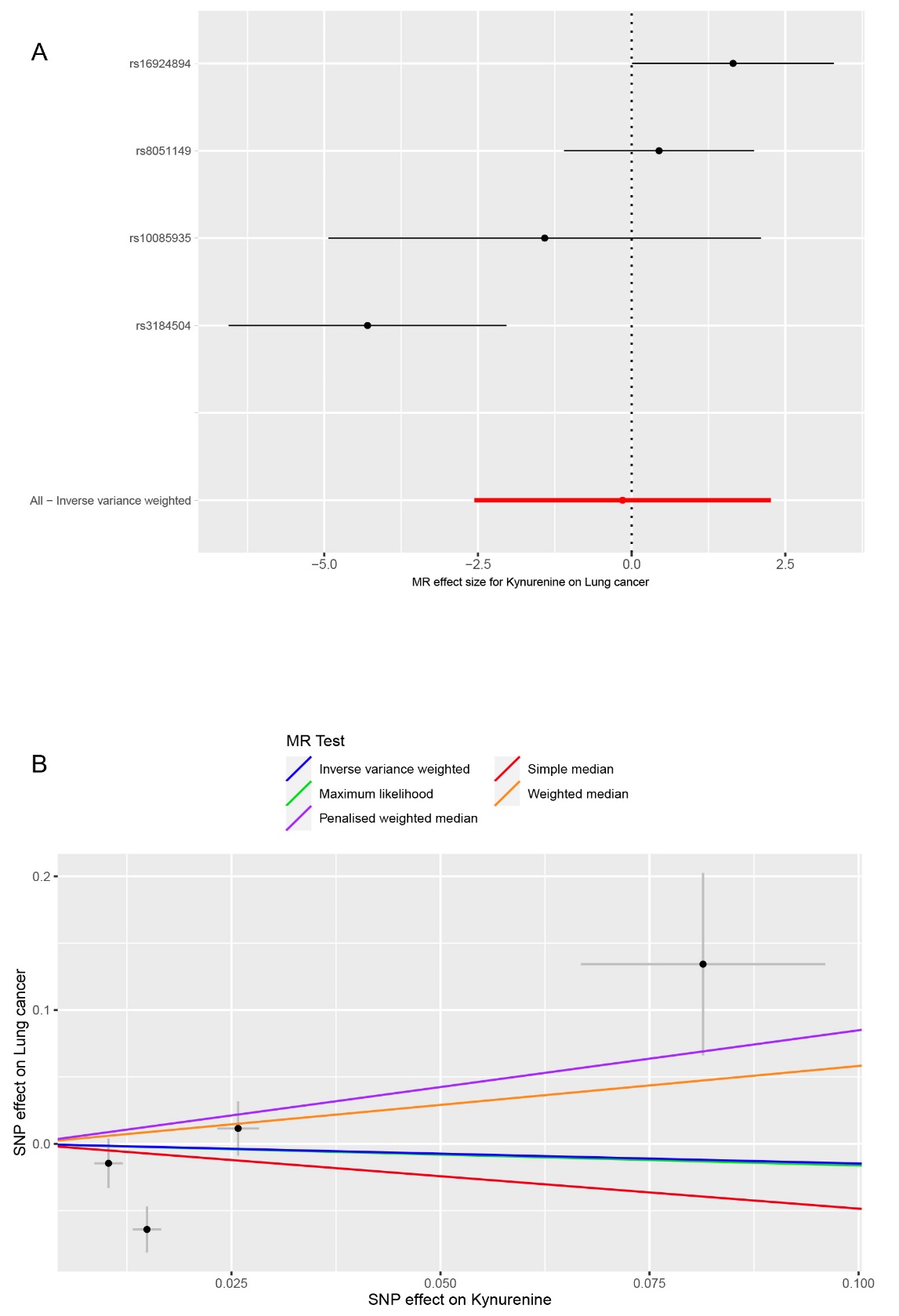


**Figure S12.** Genetically predicted associations between circulating kynurenine levels and the risk of lung cancer. A. Forest plot for the causal effects of circulating kynurenine levels on the risk of lung cancer. B. Scatter plot of different analysis methods for the causal associations between circulating kynurenine levels and the risk of lung cancer. Each approach has a different line. The slope of each line represents the causal association.


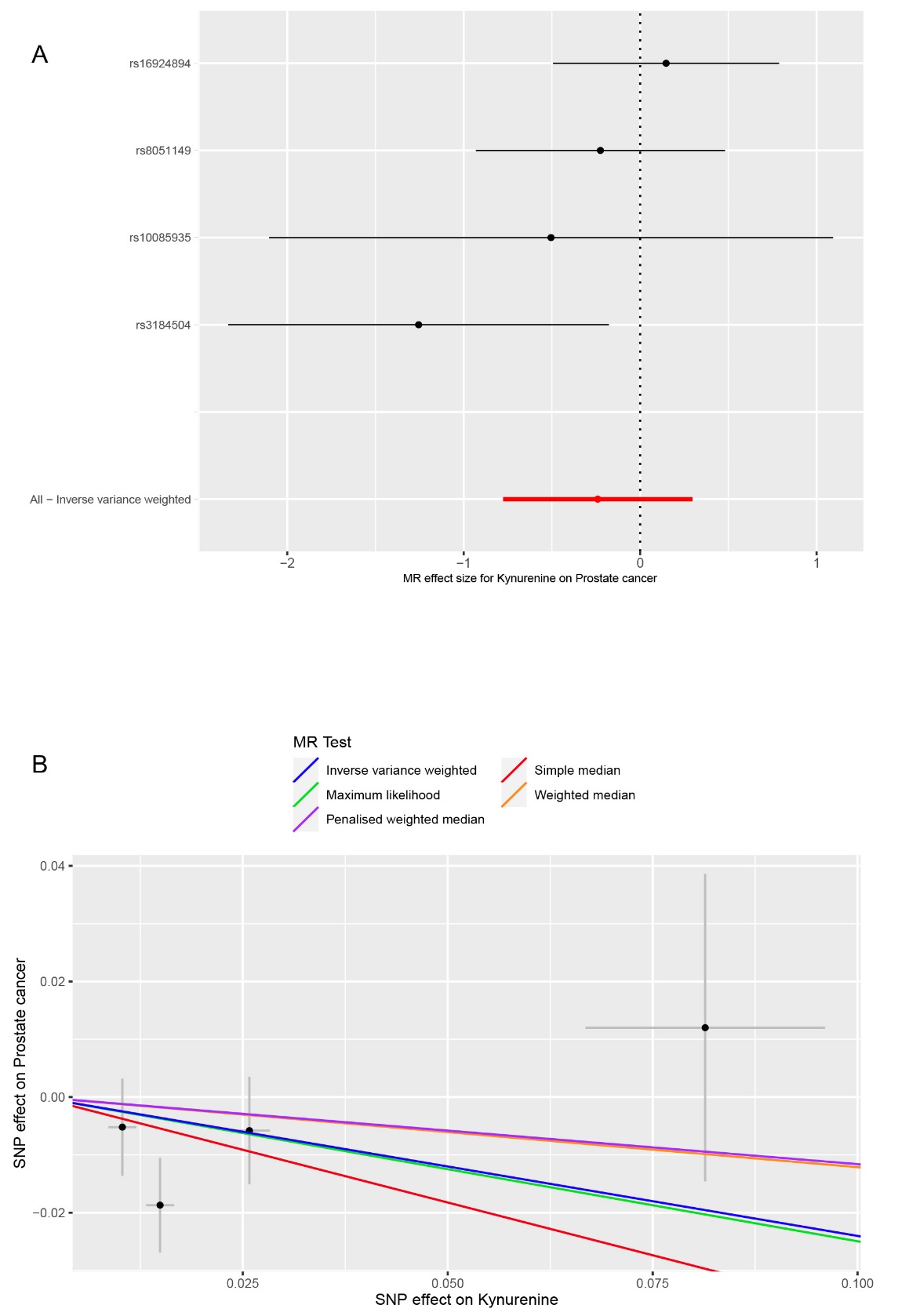


**Figure S13.** Genetically predicted associations between circulating kynurenine levels and the risk of prostate cancer. A. Forest plot for the causal effects of circulating kynurenine levels on the risk of prostate cancer. B. Scatter plot of different analysis methods for the causal associations between circulating kynurenine levels and the risk of prostate cancer. Each approach has a different line. The slope of each line represents the causal association.


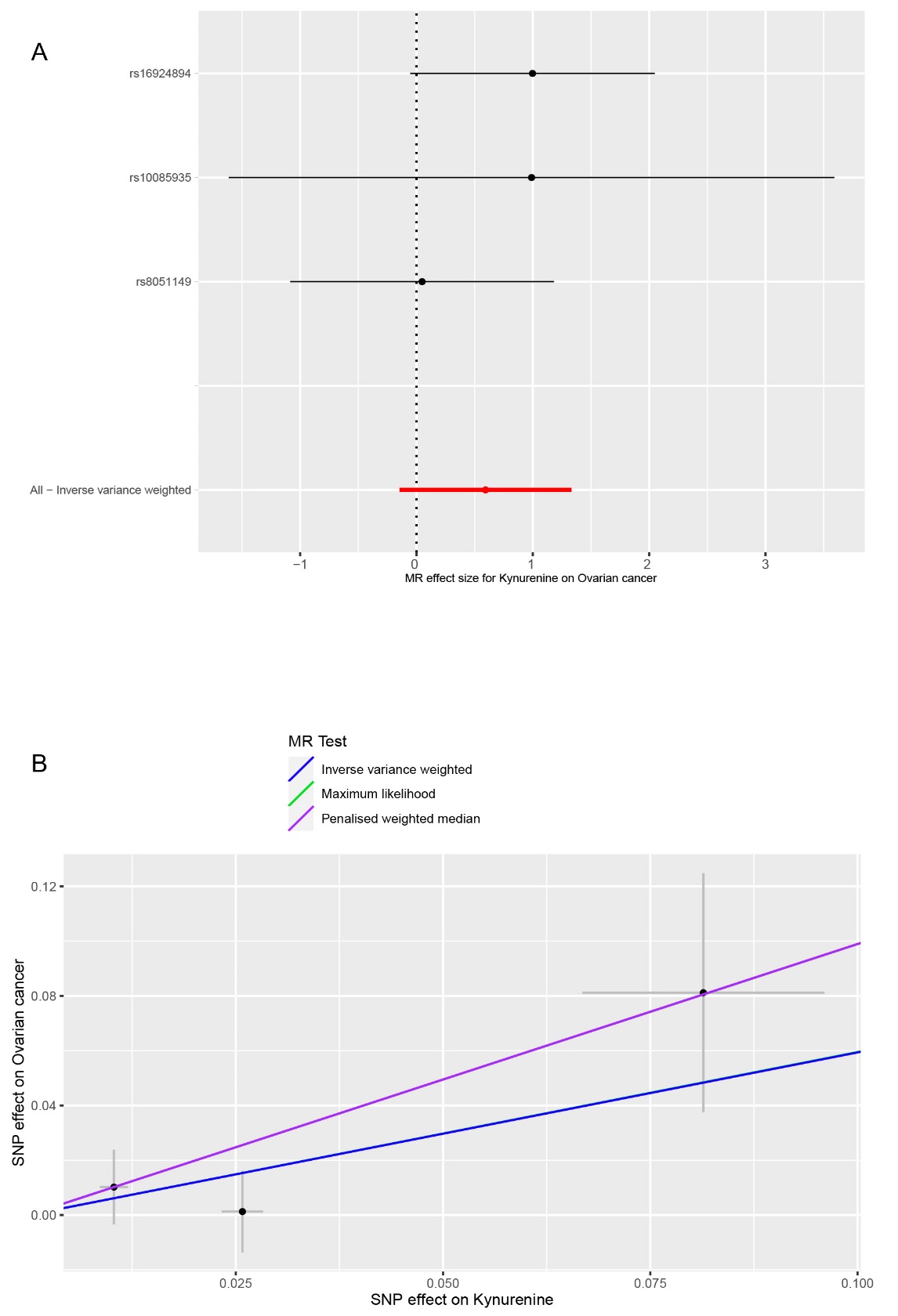


**Figure S14.** Genetically predicted associations between circulating kynurenine levels and the risk of ovarian cancer. A. Forest plot for the causal effects of circulating kynurenine levels on the risk of ovarian cancer. B. Scatter plot of different analysis methods for the causal associations between circulating kynurenine levels and the risk of ovarian cancer. Each approach has a different line. The slope of each line represents the causal association.


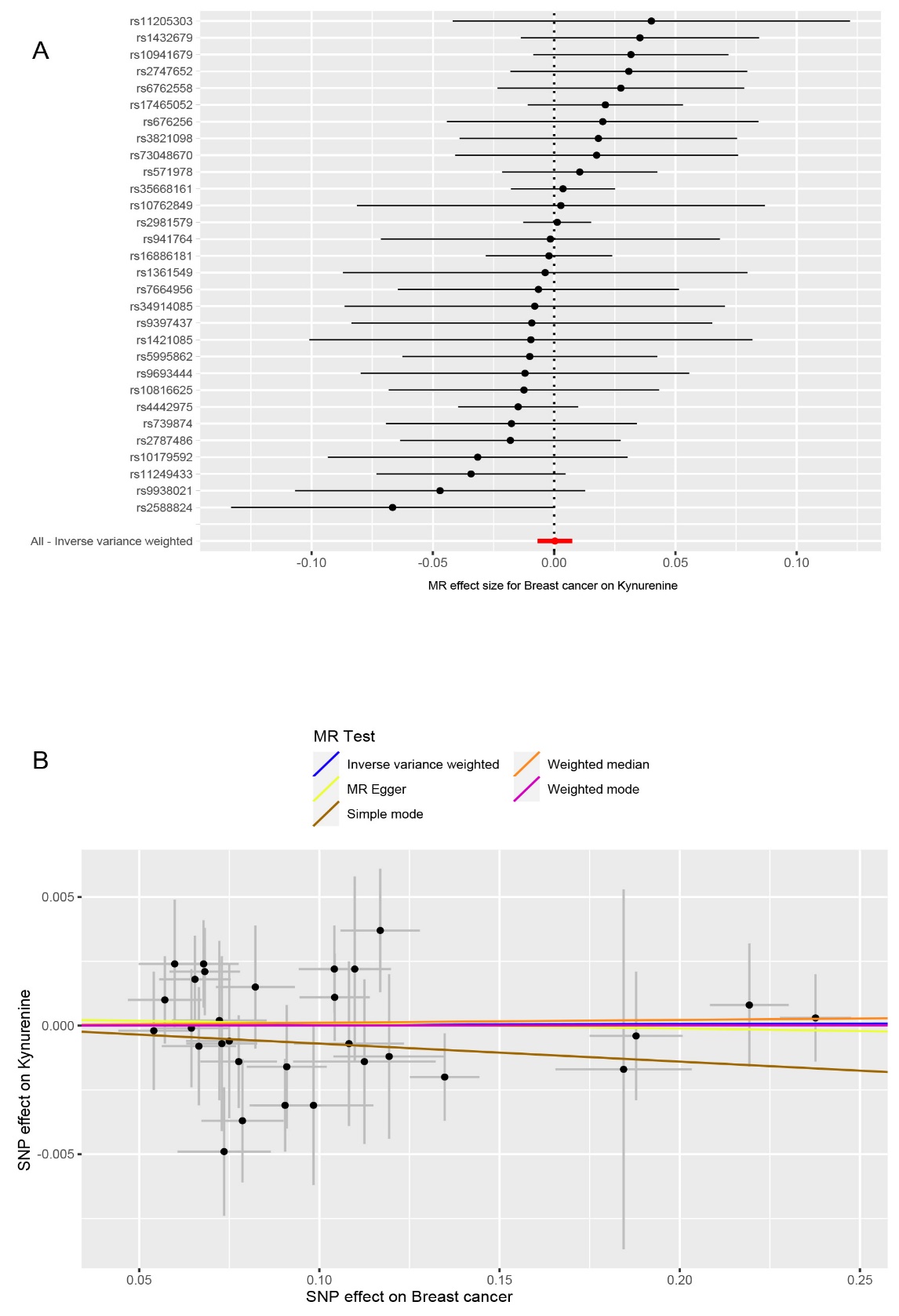


**Figure S15.** Genetically predicted associations between breast cancer and circulating kynurenine levels. A. Forest plot for the causal effects of breast cancer on circulating kynurenine levels. B. Scatter plot of different analysis methods for the causal associations between breast cancer and circulating kynurenine levels. Each approach has a different line. The slope of each line represents the causal association.


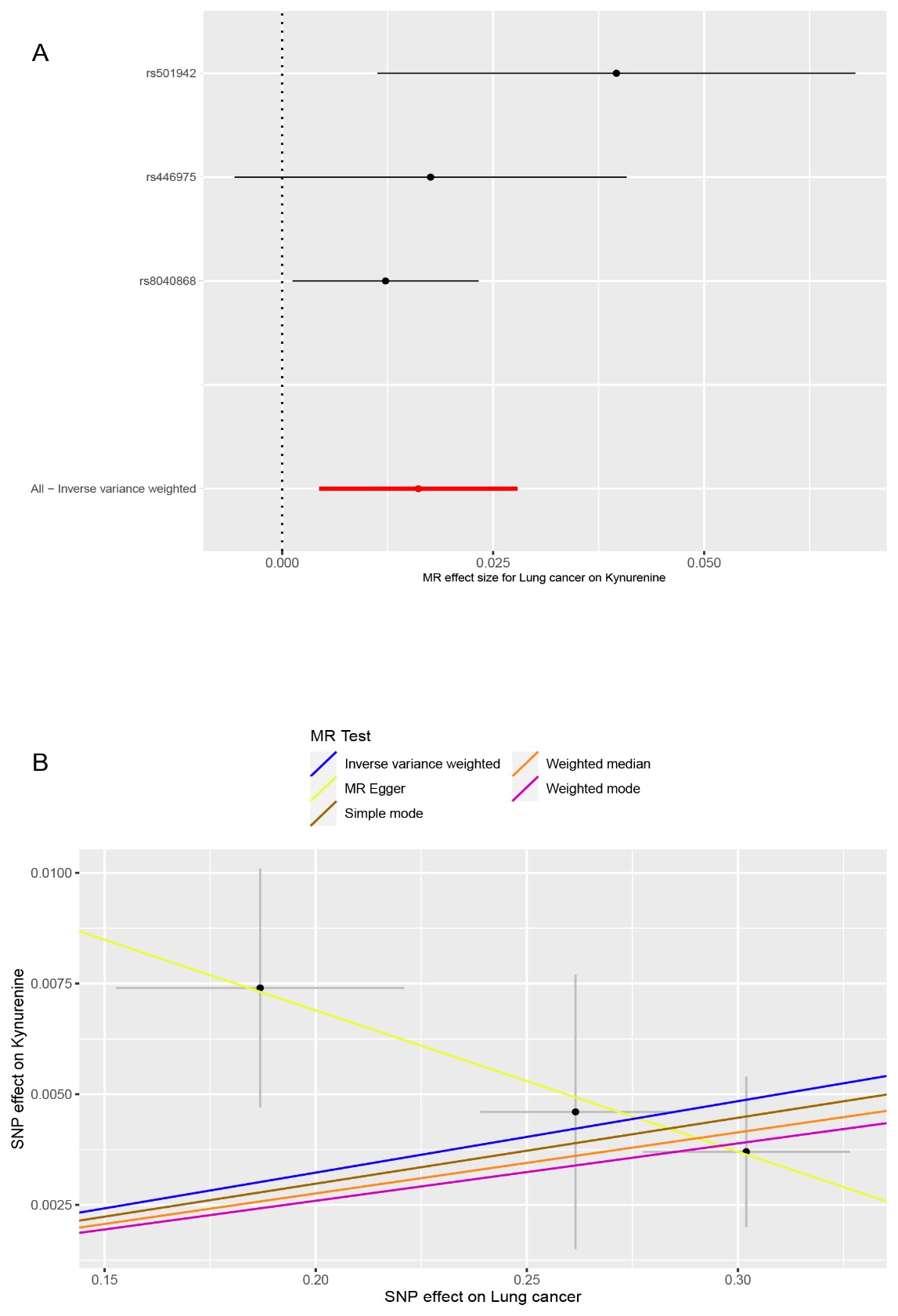


**Figure S16.** Genetically predicted associations between lung cancer and circulating kynurenine levels. A. Forest plot for the causal effects of lung cancer on circulating kynurenine levels. B. Scatter plot of different analysis methods for the causal associations between lung cancer and circulating kynurenine levels. Each approach has a different line. The slope of each line represents the causal association.


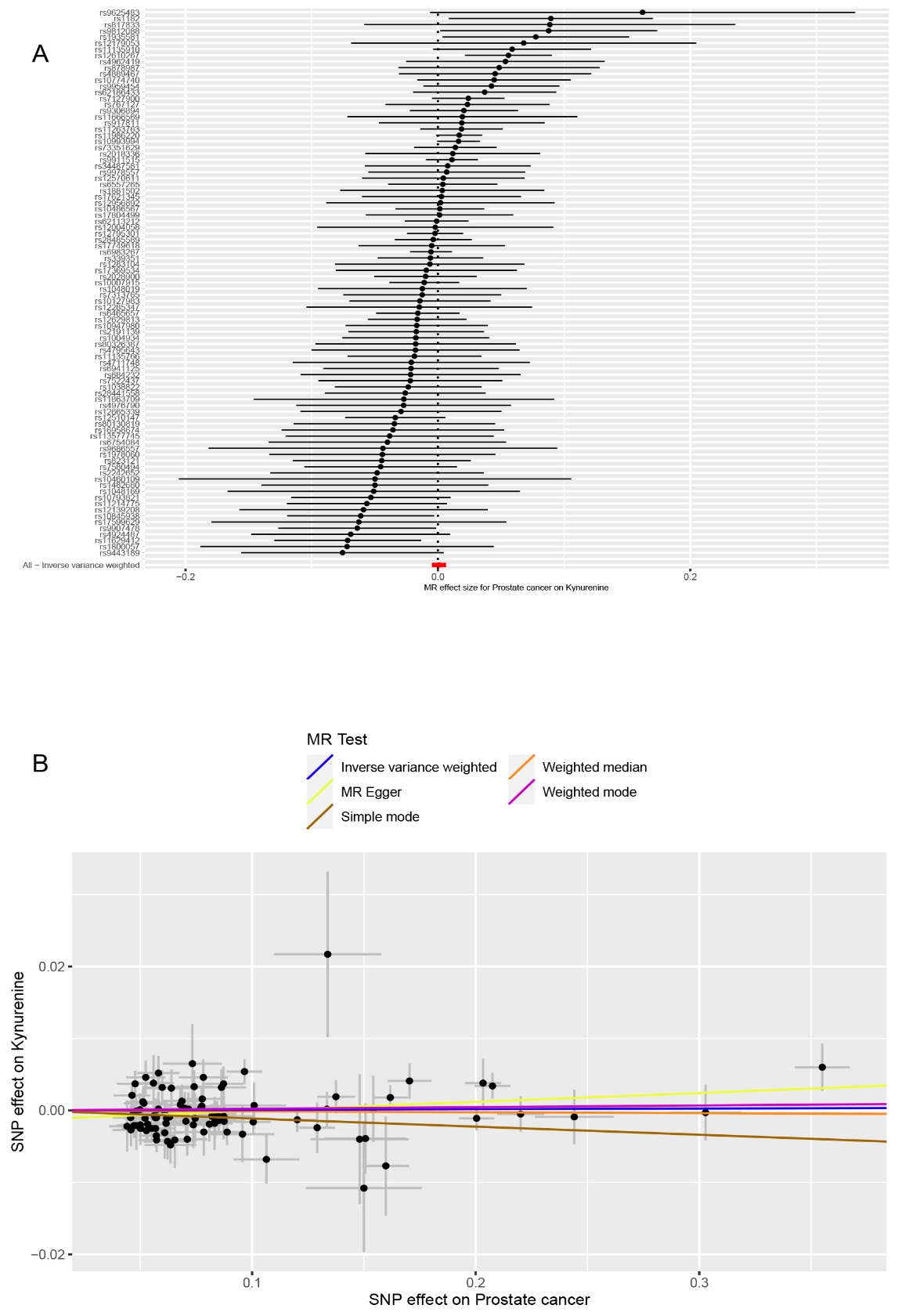


**Figure S17.** Genetically predicted associations between prostate cancer and circulating kynurenine levels. A. Forest plot for the causal effects of prostate cancer on circulating kynurenine levels. B. Scatter plot of different analysis methods for the causal associations between prostate cancer and circulating kynurenine levels. Each approach has a different line. The slope of each line represents the causal association.


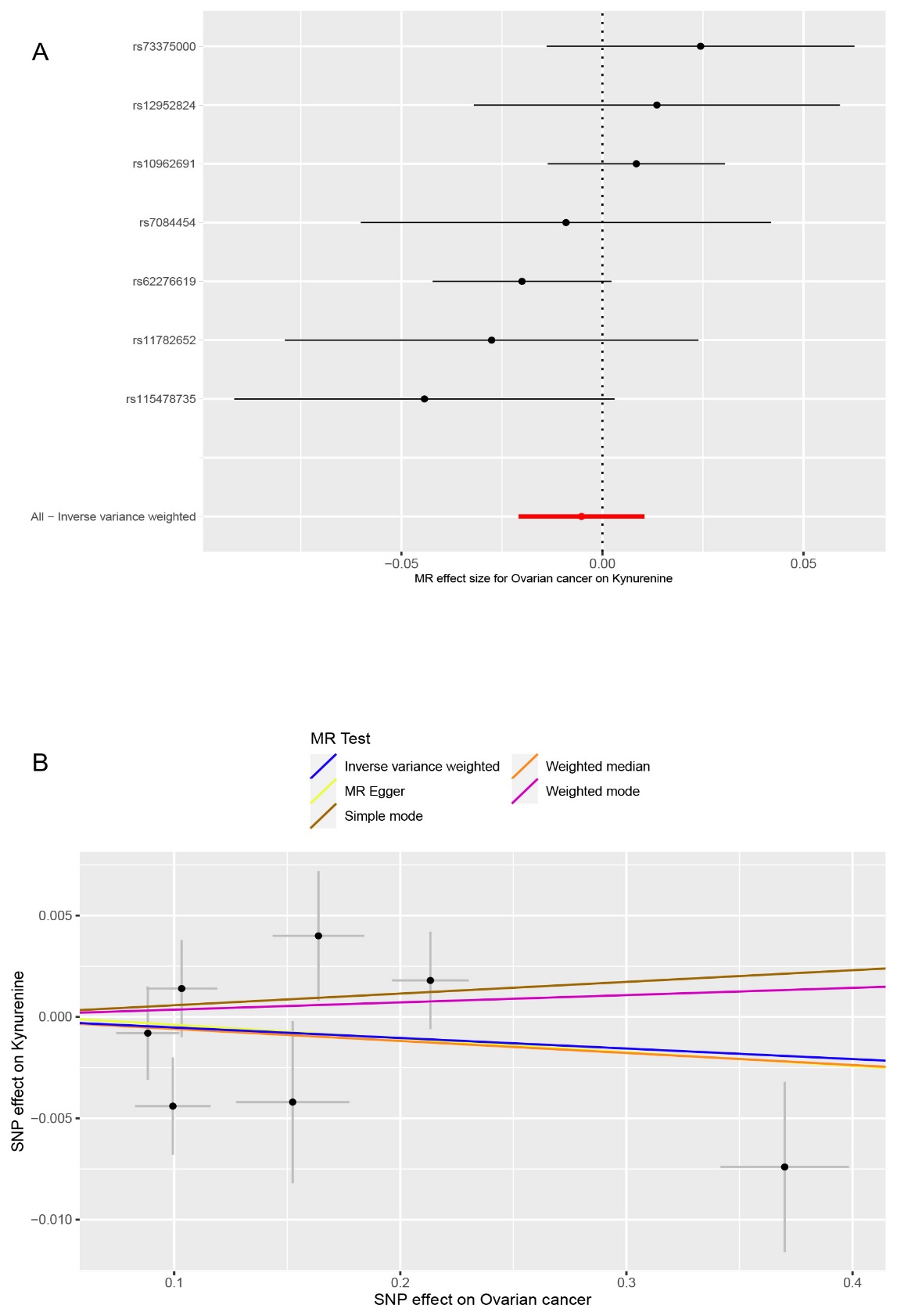


**Figure S18.** Genetically predicted associations between ovarian cancer and circulating kynurenine levels. A. Forest plot for the causal effects of ovarian cancer on circulating kynurenine levels. B. Scatter plot of different analysis methods for the causal associations between ovarian cancer and circulating kynurenine levels. Each approach has a different line. The slope of each line represents the causal association.

**2 Supplementary Tables**

| Table S1. The tryptophan-related and kynurenine-related genetic variants used for the MR analyses^a^ | | | | | | | | | | | | |
| --- | --- | --- | --- | --- | --- | --- | --- | --- | --- | --- | --- | --- |
| Exposure | SNP | Chr | Position | Effect allel | Alternative allele | EAF | Beta | SE | P value | Nearby gene | R^2^ | F-statistic |
| Tryptophan | rs13122250 | 4 | 157027738 | T | C | 0.5542 | 0.0062 | 0.0009 | 8.949E-12 | NA | 0.003005 | 47.46 |
| Tryptophan | rs1016522 | 5 | 143173519 | A | G | 0.5803 | 0.0058 | 0.0009 | 1.589E-10 | HMHB1 | 0.002592 | 41.53 |
| Tryptophan | rs4306882 | 3 | 21037588 | T | G | 0.6153 | -0.0057 | 0.0009 | 2.52E-10 | NA | 0.002433 | 40.11 |
| Tryptophan | rs6935961 | 6 | 125678049 | A | G | 0.5578 | -0.0056 | 0.0009 | 3.753E-10 | NA | 0.002447 | 38.72 |
| Tryptophan | rs284191 | 1 | 92008636 | A | G | 0.6150 | -0.0060 | 0.0010 | 1.973E-09 | TGFBR3 | 0.002184 | 36.00 |
| Tryptophan | rs7463805 | 8 | 110822848 | T | C | 0.5213 | -0.0053 | 0.0009 | 4.596E-09 | NA | 0.002218 | 34.68 |
| Tryptophan | rs1559063 | 5 | 172281200 | C | G | 0.6209 | 0.0052 | 0.0009 | 7.818E-09 | ERGIC1 | 0.002014 | 33.38 |
| Tryptophan | rs38271 | 7 | 14273556 | A | G | 0.5925 | -0.0051 | 0.0009 | 1.185E-08 | DGKB | 0.001987 | 32.11 |
| Tryptophan | rs2111118 | 16 | 52606654 | T | C | 0.6207 | 0.0051 | 0.0009 | 1.212E-08 | FTO | 0.001937 | 32.11 |
| Tryptophan | rs603446 | 11 | 116159645 | T | C | 0.4448 | 0.0051 | 0.0009 | 1.383E-08 | ZPR1 | 0.002032 | 32.11 |
| Tryptophan | rs972459 | 3 | 193338191 | T | C | 0.5787 | 0.0050 | 0.0009 | 1.969E-08 | NA | 0.001928 | 30.86 |
| Tryptophan | rs4958379 | 5 | 153990234 | A | G | 0.4330 | -0.0050 | 0.0009 | 2.176E-08 | NA | 0.001942 | 30.86 |
| Tryptophan | rs1373962 | 5 | 83563618 | T | C | 0.5973 | 0.0050 | 0.0009 | 2.707E-08 | EDIL3 | 0.001903 | 30.86 |
| Tryptophan | rs9511152 | 13 | 18494767 | A | G | 0.5590 | -0.0050 | 0.0009 | 2.985E-08 | NA | 0.001950 | 30.86 |
| Tryptophan | rs710580 | 3 | 191291857 | A | C | 0.3566 | -0.0050 | 0.0009 | 3.574E-08 | P3H2 | 0.001815 | 30.86 |
| Tryptophan | rs7584842 | 2 | 11673711 | T | C | 0.5377 | -0.0050 | 0.0009 | 4.15E-08 | GREB1 | 0.001966 | 30.86 |
| Tryptophan | rs6480970 | 10 | 54238670 | A | G | 0.6159 | -0.0049 | 0.0009 | 4.287E-08 | LOC105378306/7 | 0.001797 | 29.64 |
| Tryptophan | rs4615256 | 5 | 62736623 | A | G | 0.5327 | 0.0049 | 0.0009 | 4.988E-08 | LOC107986418 | 0.001891 | 29.64 |
| Kynurenine | rs8051149 | 16 | 86436323 | A | G | 0.2127 | 0.0258 | 0.0025 | 9.073E-26 | SLC7A5 | 0.004564 | 106.50 |
| Kynurenine | rs3184504 | 12 | 110368991 | T | C | 0.4851 | 0.0149 | 0.0017 | 6.046E-18 | SH2B3 | 0.004910 | 76.82 |
| Kynurenine | rs10085935 | 8 | 39925424 | T | C | 0.3782 | -0.0103 | 0.0017 | 3.326E-09 | IDO2 | 0.002209 | 36.71 |
| Kynurenine | rs16924894 | 10 | 24885531 | A | T | 0.0240 | 0.0814 | 0.0146 | 2.329E-08 | NA | 0.000186 | 31.08 |
| Abbreviations: SNP, single-nucleotide polymorphism; Chr, chromosome; EAF, effect allel frequency; SE, standard error. | | | | | | | | | | | | |
| Source: ^a^Shin SY, Fauman EB, Petersen AK et al. An atlas of genetic influences on human blood metabolites. Nat Genet 2014; 46: 543-550. | | | | | | | | | | | | |

| Table S2. The site-specific cancers-related instrumental variables used for the MR analyses | | | | | | | | | | | |
| --- | --- | --- | --- | --- | --- | --- | --- | --- | --- | --- | --- |
| Exposure | SNP | Chr | Position | Reference allel | Alternative allel | EAF | Beta | SE | *P* value | R^2^ | F-statistic |
| Breast cancer | rs9938021 | 16 | 80648048 | C | G | 0.2285 | 0.0787 | 0.0114 | 5.35E-12 | 0.000187 | 47.53 |
| Breast cancer | rs9693444 | 8 | 29509616 | A | C | 0.6761 | -0.0666 | 0.0103 | 1.09E-10 | 0.000203 | 41.61 |
| Breast cancer | rs941764 | 14 | 91841069 | A | G | 0.3399 | 0.0645 | 0.0102 | 2.37E-10 | 0.000201 | 40.14 |
| Breast cancer | rs9397437 | 6 | 151952332 | G | A | 0.0654 | 0.1844 | 0.0189 | 1.98E-22 | 0.000129 | 94.92 |
| Breast cancer | rs7664956 | 4 | 175832937 | G | T | 0.1201 | -0.1082 | 0.0152 | 1.24E-12 | 0.000119 | 50.42 |
| Breast cancer | rs739874 | 14 | 68974410 | A | G | 0.2670 | -0.0910 | 0.0111 | 2.67E-16 | 0.000293 | 67.02 |
| Breast cancer | rs73048670 | 19 | 44284531 | C | T | 0.3993 | 0.0571 | 0.0103 | 2.88E-08 | 0.000165 | 30.81 |
| Breast cancer | rs676256 | 9 | 110895353 | C | T | 0.6166 | 0.1098 | 0.0101 | 1.00E-27 | 0.000628 | 119.15 |
| Breast cancer | rs6762558 | 3 | 4742251 | A | G | 0.3959 | 0.0655 | 0.0099 | 4.44E-11 | 0.000231 | 43.38 |
| Breast cancer | rs5995862 | 22 | 40857793 | A | C | 0.1045 | 0.1194 | 0.0155 | 1.31E-14 | 0.000124 | 59.38 |
| Breast cancer | rs571978 | 3 | 27344193 | A | G | 0.5223 | 0.1043 | 0.0097 | 9.55E-27 | 0.000637 | 114.56 |
| Breast cancer | rs4442975 | 2 | 217920769 | G | T | 0.5061 | -0.1348 | 0.0097 | 2.91E-44 | 0.001085 | 194.67 |
| Breast cancer | rs3821098 | 2 | 218292141 | T | C | 0.7370 | -0.0822 | 0.0109 | 5.63E-14 | 0.000244 | 56.51 |
| Breast cancer | rs35668161 | 16 | 52538825 | C | A | 0.2492 | 0.2193 | 0.0109 | 1.71E-89 | 0.001680 | 402.60 |
| Breast cancer | rs34914085 | 14 | 37128564 | C | A | 0.2098 | -0.0750 | 0.0120 | 4.23E-10 | 0.000144 | 38.99 |
| Breast cancer | rs2981579 | 10 | 123337335 | A | G | 0.5956 | -0.2377 | 0.0099 | 1.29E-128 | 0.003127 | 582.21 |
| Breast cancer | rs2787486 | 17 | 53209774 | A | C | 0.3008 | -0.0776 | 0.0107 | 3.09E-13 | 0.000249 | 53.12 |
| Breast cancer | rs2747652 | 6 | 152437016 | T | C | 0.5324 | 0.0682 | 0.0098 | 2.97E-12 | 0.000271 | 48.74 |
| Breast cancer | rs2588824 | 14 | 68619461 | T | C | 0.8354 | -0.0736 | 0.0130 | 1.33E-08 | 0.000099 | 32.27 |
| Breast cancer | rs17465052 | 8 | 128354080 | G | A | 0.4246 | -0.1042 | 0.0099 | 6.31E-26 | 0.000604 | 110.92 |
| Breast cancer | rs16886181 | 5 | 56029243 | T | C | 0.1586 | 0.1879 | 0.0129 | 6.32E-48 | 0.000629 | 211.49 |
| Breast cancer | rs1432679 | 5 | 158244083 | C | T | 0.5679 | -0.0679 | 0.0098 | 3.60E-12 | 0.000265 | 48.36 |
| Breast cancer | rs1421085 | 16 | 53800954 | T | C | 0.4131 | -0.0729 | 0.0099 | 1.39E-13 | 0.000296 | 54.72 |
| Breast cancer | rs1361549 | 6 | 82287685 | G | A | 0.4250 | -0.0541 | 0.0098 | 3.80E-08 | 0.000165 | 30.23 |
| Breast cancer | rs11249433 | 1 | 121280613 | A | G | 0.4018 | 0.0905 | 0.0099 | 4.43E-20 | 0.000451 | 84.23 |
| Breast cancer | rs11205303 | 1 | 149906413 | T | C | 0.3984 | 0.0599 | 0.0100 | 2.36E-09 | 0.000190 | 35.62 |
| Breast cancer | rs10941679 | 5 | 44706498 | A | G | 0.2543 | 0.1169 | 0.0110 | 3.20E-26 | 0.000475 | 112.33 |
| Breast cancer | rs10816625 | 9 | 110837073 | A | G | 0.0610 | 0.1125 | 0.0198 | 1.32E-08 | 0.000041 | 32.32 |
| Breast cancer | rs10762849 | 10 | 80886061 | C | T | 0.1621 | 0.0723 | 0.0131 | 3.77E-08 | 0.000092 | 30.28 |
| Breast cancer | rs10179592 | 2 | 121246568 | T | C | 0.9015 | 0.0984 | 0.0166 | 3.35E-09 | 0.000069 | 34.95 |
| Lung cancer | rs8040868 | 15 | 78911181 | T | C | 0.424371 | 0.301974 | 0.024535 | 4.97E-60 | 0.002720 | 151.48 |
| Lung cancer | rs446975 | 3 | 9217383 | G | T | 0.115137 | -0.261532 | 0.022649 | 6.42E-18 | 0.000999 | 133.34 |
| Lung cancer | rs501942 | 6 | 31840477 | C | T | 0.108859 | 0.186841 | 0.034137 | 1.47E-10 | 0.000214 | 29.96 |
| Prostate cancer | rs9978557 | 21 | 42882462 | C | T | 0.098 | -0.1009 | 0.0143 | 2.02E-12 | 0.000063 | 49.79 |
| Prostate cancer | rs9959454 | 18 | 76770820 | A | G | 0.2672 | -0.0872 | 0.009 | 5.34E-22 | 0.000262 | 93.87 |
| Prostate cancer | rs9911515 | 17 | 69115358 | A | G | 0.5197 | -0.1618 | 0.0079 | 6.47E-93 | 0.001493 | 419.47 |
| Prostate cancer | rs9907478 | 17 | 47395729 | C | T | 0.0757 | 0.1064 | 0.0147 | 4.52E-13 | 0.000052 | 52.39 |
| Prostate cancer | rs9812088 | 3 | 128263668 | G | A | 0.3 | -0.0524 | 0.0088 | 2.99E-09 | 0.000106 | 35.46 |
| Prostate cancer | rs9686557 | 5 | 172959030 | A | C | 0.444 | 0.0482 | 0.008 | 1.94E-09 | 0.000128 | 36.30 |
| Prostate cancer | rs9625483 | 22 | 28888939 | G | A | 0.0288 | 0.1338 | 0.024 | 2.43E-08 | 0.000012 | 31.08 |
| Prostate cancer | rs9443189 | 6 | 76495882 | A | G | 0.1429 | -0.0635 | 0.0116 | 4.68E-08 | 0.000052 | 29.97 |
| Prostate cancer | rs9306894 | 2 | 20878105 | A | G | 0.3633 | 0.0777 | 0.0082 | 1.92E-21 | 0.000296 | 89.79 |
| Prostate cancer | rs917811 | 7 | 21059022 | C | T | 0.6856 | -0.0686 | 0.0085 | 5.51E-16 | 0.000200 | 65.13 |
| Prostate cancer | rs878987 | 11 | 134266372 | A | G | 0.1459 | 0.0639 | 0.0117 | 4.77E-08 | 0.000053 | 29.83 |
| Prostate cancer | rs823121 | 1 | 205724302 | G | A | 0.4322 | -0.0472 | 0.008 | 3.38E-09 | 0.000122 | 34.81 |
| Prostate cancer | rs817833 | 9 | 110152977 | G | T | 0.8914 | -0.0733 | 0.0131 | 2.14E-08 | 0.000043 | 31.31 |
| Prostate cancer | rs80326387 | 15 | 66705043 | G | A | 0.2583 | 0.0567 | 0.0091 | 4.64E-10 | 0.000106 | 38.82 |
| Prostate cancer | rs80130819 | 12 | 48419618 | A | C | 0.0916 | -0.0957 | 0.0142 | 1.89E-11 | 0.000054 | 45.42 |
| Prostate cancer | rs767127 | 14 | 69134264 | A | G | 0.4989 | 0.0512 | 0.0079 | 1.00E-10 | 0.000150 | 42.00 |
| Prostate cancer | rs7580494 | 2 | 62781499 | C | T | 0.3878 | 0.0551 | 0.0081 | 1.17E-11 | 0.000157 | 46.27 |
| Prostate cancer | rs7522437 | 1 | 204029890 | G | A | 0.4609 | -0.0457 | 0.008 | 1.21E-08 | 0.000116 | 32.63 |
| Prostate cancer | rs73351629 | 8 | 128018466 | C | G | 0.3334 | -0.1375 | 0.0085 | 5.15E-59 | 0.000829 | 261.68 |
| Prostate cancer | rs7313765 | 12 | 65011980 | G | A | 0.4218 | -0.0563 | 0.0082 | 5.72E-12 | 0.000164 | 47.14 |
| Prostate cancer | rs7127900 | 11 | 2233574 | A | G | 0.8015 | -0.1704 | 0.0098 | 4.94E-68 | 0.000686 | 302.33 |
| Prostate cancer | rs6983267 | 8 | 128413305 | G | T | 0.4892 | -0.2004 | 0.0079 | 2.81E-141 | 0.002293 | 643.49 |
| Prostate cancer | rs6941125 | 6 | 109287209 | T | A | 0.1457 | 0.0704 | 0.0111 | 1.99E-10 | 0.000071 | 40.23 |
| Prostate cancer | rs684232 | 17 | 618965 | T | C | 0.3534 | 0.0832 | 0.0082 | 4.34E-24 | 0.000335 | 102.95 |
| Prostate cancer | rs6754084 | 2 | 202124997 | T | C | 0.7324 | 0.05 | 0.0091 | 3.53E-08 | 0.000084 | 30.19 |
| Prostate cancer | rs6557265 | 6 | 153433402 | T | C | 0.4226 | -0.077 | 0.008 | 1.04E-21 | 0.000322 | 92.64 |
| Prostate cancer | rs6465657 | 7 | 97816327 | C | T | 0.5365 | -0.1005 | 0.0079 | 6.96E-37 | 0.000574 | 161.84 |
| Prostate cancer | rs62186433 | 2 | 242257835 | A | G | 0.1486 | 0.0863 | 0.011 | 3.21E-15 | 0.000111 | 61.55 |
| Prostate cancer | rs62113212 | 19 | 51360840 | C | T | 0.0775 | -0.3028 | 0.0159 | 4.26E-81 | 0.000370 | 362.67 |
| Prostate cancer | rs4976790 | 5 | 177968915 | G | T | 0.1131 | 0.0737 | 0.0127 | 6.73E-09 | 0.000048 | 33.68 |
| Prostate cancer | rs4962419 | 10 | 126697114 | G | A | 0.2664 | 0.0598 | 0.0089 | 1.62E-11 | 0.000126 | 45.15 |
| Prostate cancer | rs4924487 | 15 | 40922915 | G | C | 0.8359 | 0.0622 | 0.0109 | 1.32E-08 | 0.000064 | 32.56 |
| Prostate cancer | rs4889467 | 16 | 82169385 | C | T | 0.6506 | -0.0463 | 0.0082 | 1.91E-08 | 0.000103 | 31.88 |
| Prostate cancer | rs4795643 | 17 | 30092614 | C | A | 0.2287 | -0.0618 | 0.0103 | 1.64E-09 | 0.000091 | 36.00 |
| Prostate cancer | rs4711748 | 6 | 43694598 | T | C | 0.7746 | -0.0521 | 0.0094 | 3.36E-08 | 0.000076 | 30.72 |
| Prostate cancer | rs34487581 | 10 | 897201 | G | A | 0.1585 | 0.0775 | 0.0109 | 9.34E-13 | 0.000096 | 50.55 |
| Prostate cancer | rs339351 | 6 | 117200434 | C | A | 0.3052 | -0.0843 | 0.0087 | 2.90E-22 | 0.000284 | 93.89 |
| Prostate cancer | rs28485589 | 2 | 173303031 | A | G | 0.0608 | -0.2441 | 0.0176 | 1.11E-43 | 0.000157 | 192.36 |
| Prostate cancer | rs28441558 | 17 | 7803118 | T | C | 0.0548 | 0.1507 | 0.0182 | 1.02E-16 | 0.000051 | 68.56 |
| Prostate cancer | rs2242652 | 5 | 1280028 | G | A | 0.2054 | -0.1598 | 0.0105 | 3.46E-52 | 0.000539 | 231.62 |
| Prostate cancer | rs2191139 | 19 | 42001210 | T | C | 0.7474 | 0.0875 | 0.0093 | 3.25E-21 | 0.000238 | 88.52 |
| Prostate cancer | rs2028900 | 2 | 85767735 | C | T | 0.4418 | -0.082 | 0.008 | 6.67E-25 | 0.000369 | 105.06 |
| Prostate cancer | rs2018336 | 6 | 11217897 | T | C | 0.2206 | -0.0679 | 0.0096 | 1.91E-12 | 0.000123 | 50.03 |
| Prostate cancer | rs1978060 | 22 | 19749525 | A | G | 0.6127 | 0.0568 | 0.0083 | 8.54E-12 | 0.000158 | 46.83 |
| Prostate cancer | rs1935581 | 10 | 90195149 | C | T | 0.3728 | -0.0477 | 0.0082 | 6.55E-09 | 0.000113 | 33.84 |
| Prostate cancer | rs1881502 | 11 | 1507512 | T | C | 0.8097 | -0.0581 | 0.0101 | 7.42E-09 | 0.000073 | 33.09 |
| Prostate cancer | rs1800057 | 11 | 108143456 | C | G | 0.0234 | 0.15 | 0.026 | 8.15E-09 | 0.000011 | 33.28 |
| Prostate cancer | rs17804499 | 4 | 74442349 | G | C | 0.0534 | -0.1542 | 0.0192 | 9.15E-16 | 0.000046 | 64.50 |
| Prostate cancer | rs17749618 | 11 | 76251818 | A | G | 0.324 | -0.0609 | 0.0085 | 7.48E-13 | 0.000160 | 51.33 |
| Prostate cancer | rs17621345 | 7 | 40875192 | A | C | 0.2587 | -0.0715 | 0.0095 | 6.72E-14 | 0.000155 | 56.65 |
| Prostate cancer | rs17599629 | 1 | 150658287 | A | G | 0.2181 | 0.0654 | 0.0096 | 9.87E-12 | 0.000113 | 46.41 |
| Prostate cancer | rs17369534 | 3 | 152064639 | G | C | 0.1047 | -0.0873 | 0.0134 | 6.51E-11 | 0.000057 | 42.44 |
| Prostate cancer | rs16958674 | 16 | 57693055 | C | T | 0.2385 | -0.0533 | 0.0094 | 1.27E-08 | 0.000083 | 32.15 |
| Prostate cancer | rs1482680 | 5 | 44392142 | G | A | 0.6667 | -0.0501 | 0.0085 | 3.58E-09 | 0.000110 | 34.74 |
| Prostate cancer | rs12956892 | 18 | 56746315 | G | T | 0.3021 | 0.0498 | 0.0086 | 7.68E-09 | 0.000101 | 33.53 |
| Prostate cancer | rs1283104 | 3 | 106962521 | C | G | 0.3788 | 0.047 | 0.0082 | 8.81E-09 | 0.000110 | 32.85 |
| Prostate cancer | rs12795301 | 11 | 68992285 | C | A | 0.1586 | 0.2201 | 0.0105 | 3.07E-98 | 0.000836 | 439.40 |
| Prostate cancer | rs12665339 | 6 | 30601232 | A | G | 0.1674 | 0.0615 | 0.0106 | 5.56E-09 | 0.000067 | 33.66 |
| Prostate cancer | rs12629813 | 3 | 113284149 | C | T | 0.4371 | -0.0851 | 0.008 | 1.98E-26 | 0.000397 | 113.16 |
| Prostate cancer | rs12610267 | 19 | 38744733 | A | G | 0.5103 | -0.0966 | 0.0079 | 4.87E-34 | 0.000533 | 149.52 |
| Prostate cancer | rs12570611 | 10 | 104421679 | G | C | 0.3242 | -0.0699 | 0.0085 | 2.54E-16 | 0.000211 | 67.63 |
| Prostate cancer | rs12510147 | 4 | 95521863 | G | A | 0.3115 | -0.0888 | 0.0086 | 4.34E-25 | 0.000326 | 106.62 |
| Prostate cancer | rs12285347 | 11 | 102396607 | T | C | 0.4507 | -0.0745 | 0.0082 | 1.17E-19 | 0.000291 | 82.54 |
| Prostate cancer | rs12179053 | 6 | 160711566 | C | T | 0.2603 | -0.0559 | 0.0091 | 6.50E-10 | 0.000104 | 37.73 |
| Prostate cancer | rs12139208 | 1 | 88213014 | T | C | 0.6286 | -0.0458 | 0.0082 | 2.58E-08 | 0.000104 | 31.20 |
| Prostate cancer | rs12004058 | 9 | 18564470 | C | G | 0.312 | -0.0481 | 0.0086 | 2.36E-08 | 0.000096 | 31.28 |
| Prostate cancer | rs11986220 | 8 | 128531689 | A | T | 0.8953 | -0.355 | 0.0122 | 1.10E-187 | 0.001132 | 846.71 |
| Prostate cancer | rs11863709 | 16 | 57654576 | C | T | 0.0403 | -0.1481 | 0.022 | 1.78E-11 | 0.000025 | 45.32 |
| Prostate cancer | rs1182 | 9 | 132576060 | C | A | 0.2198 | 0.0581 | 0.0095 | 1.10E-09 | 0.000091 | 37.40 |
| Prostate cancer | rs11666569 | 19 | 17214073 | C | T | 0.2867 | -0.0516 | 0.009 | 8.17E-09 | 0.000096 | 32.87 |
| Prostate cancer | rs11629412 | 14 | 37138294 | G | C | 0.5821 | 0.0573 | 0.0082 | 2.34E-12 | 0.000169 | 48.83 |
| Prostate cancer | rs113577745 | 2 | 10135681 | C | G | 0.098 | 0.0784 | 0.0131 | 2.09E-09 | 0.000045 | 35.82 |
| Prostate cancer | rs11263763 | 17 | 36103565 | G | A | 0.5292 | 0.2033 | 0.008 | 3.06E-141 | 0.002294 | 645.80 |
| Prostate cancer | rs11214775 | 11 | 113807181 | G | A | 0.2915 | -0.071 | 0.009 | 3.93E-15 | 0.000183 | 62.23 |
| Prostate cancer | rs11135910 | 8 | 25892142 | C | T | 0.1529 | 0.0782 | 0.011 | 9.19E-13 | 0.000093 | 50.54 |
| Prostate cancer | rs11135766 | 8 | 23533623 | C | T | 0.5736 | -0.1291 | 0.008 | 5.09E-59 | 0.000908 | 260.42 |
| Prostate cancer | rs10993994 | 10 | 51549496 | T | C | 0.617 | -0.2075 | 0.008 | 2.29E-147 | 0.002267 | 672.75 |
| Prostate cancer | rs10947980 | 6 | 41525739 | A | G | 0.2634 | 0.0834 | 0.0089 | 8.78E-21 | 0.000243 | 87.81 |
| Prostate cancer | rs10845938 | 12 | 14416918 | A | G | 0.5541 | 0.0572 | 0.008 | 9.80E-13 | 0.000180 | 51.12 |
| Prostate cancer | rs10793821 | 5 | 133836209 | C | T | 0.5795 | 0.0527 | 0.008 | 5.43E-11 | 0.000151 | 43.40 |
| Prostate cancer | rs10774740 | 12 | 114666202 | G | T | 0.3844 | -0.0741 | 0.0082 | 1.63E-19 | 0.000276 | 81.66 |
| Prostate cancer | rs10486567 | 7 | 27976563 | G | A | 0.2373 | -0.1335 | 0.0094 | 2.04E-45 | 0.000521 | 201.70 |
| Prostate cancer | rs1048169 | 9 | 19055965 | T | C | 0.379 | 0.0609 | 0.0081 | 6.53E-14 | 0.000190 | 56.53 |
| Prostate cancer | rs1048019 | 6 | 160147630 | A | G | 0.2849 | -0.0569 | 0.0091 | 3.22E-10 | 0.000114 | 39.10 |
| Prostate cancer | rs10460109 | 18 | 73036165 | T | C | 0.5857 | -0.0441 | 0.008 | 3.48E-08 | 0.000105 | 30.39 |
| Prostate cancer | rs1038822 | 2 | 43738173 | T | C | 0.6877 | -0.0809 | 0.0085 | 1.48E-21 | 0.000277 | 90.59 |
| Prostate cancer | rs10127983 | 1 | 153923276 | C | T | 0.3117 | 0.0631 | 0.0085 | 1.02E-13 | 0.000169 | 55.11 |
| Prostate cancer | rs1004934 | 10 | 122796182 | G | C | 0.3874 | 0.0572 | 0.0081 | 1.39E-12 | 0.000169 | 49.87 |
| Prostate cancer | rs10007915 | 4 | 106065308 | C | G | 0.4093 | -0.1202 | 0.0081 | 8.27E-50 | 0.000759 | 220.21 |
| Ovarian cancer | rs10962691 | 9 | 16915105 | C | G | 0.2026 | -0.2133 | 0.01698 | 1.73E-36 | 0.000767 | 157.80 |
| Ovarian cancer | rs115478735 | 9 | 136149711 | A | T | 0.1936 | 0.09941 | 0.01672 | 2.80E-09 | 0.000166 | 35.35 |
| Ovarian cancer | rs11782652 | 8 | 82653644 | A | G | 0.06811 | 0.1524 | 0.02508 | 1.25E-09 | 0.000071 | 36.92 |
| Ovarian cancer | rs12952824 | 17 | 46478195 | C | A | 0.2171 | 0.1033 | 0.0157 | 4.91E-11 | 0.000221 | 43.29 |
| Ovarian cancer | rs62276619 | 3 | 156397692 | C | T | 0.04882 | 0.37 | 0.0284 | 5.53E-39 | 0.000237 | 169.73 |
| Ovarian cancer | rs7084454 | 10 | 21821274 | G | A | 0.3279 | 0.08833 | 0.01395 | 2.41E-10 | 0.000266 | 40.09 |
| Ovarian cancer | rs73375000 | 8 | 129561866 | C | T | 0.1324 | -0.1638 | 0.02023 | 4.58E-16 | 0.000227 | 65.56 |

| Table S3. Summary-level descriptives for tryptophan and kynurenine of the KORA-TwinsUK studies^a^ | | | | | | | | | | | | | | | | | | |
| --- | --- | --- | --- | --- | --- | --- | --- | --- | --- | --- | --- | --- | --- | --- | --- | --- | --- | --- |
| Metabolite | Pathway | Super-pathway | Platform | CV(%) | Included in GWAS meta-analysis | Total N | TwinsUK | | | | | KORA | | | | | First author (year) | Web URL |
|  |  |  |  |  |  |  | N | Min | Max | Mean | SD | N | Min | Max | Mean | SD |  |  |
| Tryptophan | Tryptophan metabolism | Amino acid | LC/MS pos | 6.4 | Yes | 7,804 | 6,041 | -0.285 | 0.279 | 0 | 0.07 | 1,763 | -0.257 | 0.219 | -0.002 | 0.062 | Shin (2014) | http://metabolomics.helmholtz-muenchen.de/gwas/index.php?task=download |
| Kynurenine | Tryptophan metabolism | Amino acid | LC/MS pos | 7.3 | Yes | 7,816 | 6,052 | -0.412 | 0.429 | 0.003 | 0.113 | 1,764 | -0.42 | 0.418 | 0.002 | 0.105 | Shin (2014) | http://metabolomics.helmholtz-muenchen.de/gwas/index.php?task=download |
| Source: ^a^Shin SY, Fauman EB, Petersen AK et al. An atlas of genetic influences on human blood metabolites. Nat Genet 2014; 46: 543-550. | | | | | | | | | | | | | | | | | | |

| Table S4. Characteristics of population in the KORA-TwinsUK studies^a^ | | |
| --- | --- | --- |
| Cohort | TwinsUK | KORA |
| Country | UK | Germany |
| Gender(M/F) | 6,056 (433/5,623) | 1,768 (858/910) |
| Age (years, mean (SD)) | 53.4 (14.0) | 60.8 (8.8) |
| BMI (kg/m2, mean (SD)) | 26.1 (4.9) | 28.2 (4.8) |
| Source: ^a^Shin SY, Fauman EB, Petersen AK et al. An atlas of genetic influences on human blood metabolites. Nat Genet 2014; 46: 543-550. | | |

| Table S5. Characteristics of population related to the site-specific cancers | | | | | | | |
| --- | --- | --- | --- | --- | --- | --- | --- |
| Study/Consortium | Exposure/Outcome | Cases | Controls | Sample size | Population | First author (year) | PMID |
| BCAC | Breast cancer | 46785 | 42892 | 89677 | European | Michailidou et al., 2017 | 29059683 |
| ILCCO | Lung cancer | 11348 | 15861 | 27209 | European | Wang et al., 2014 | 24880342 |
| PRACTICAL | Prostate cancer | 79148 | 61106 | 140254 | European | Schumacher et al., 2018 | 29892016 |
| OCAC | Ovarian cancer | 25509 | 40941 | 66450 | European | Phelan et al., 2017 | 28346442 |

| Table S6. Previous studies verifing the instrumental variables strongly associated with tryptophan/kynurenine | | | | | | | | | | | |
| --- | --- | --- | --- | --- | --- | --- | --- | --- | --- | --- | --- |
| Exposure | SNP | Chr | Position | Effect allel | Other allel | EAF | Beta | SE | *P* value | Nearby gene | Study |
| Tryptophan | rs13122250 | 4 | 157027738 | T | C | 0.5542 | 0.0062 | 0.0009 | 8.949E-12 | NA | Li et al., 2019, Cheng et al., 2020 |
| Tryptophan | rs4306882 | 3 | 21037588 | T | G | 0.6153 | -0.0057 | 0.0009 | 2.52E-10 | NA | Li et al., 2019, Cheng et al., 2020 |
| Tryptophan | rs6935961 | 6 | 125678049 | A | G | 0.5578 | -0.0056 | 0.0009 | 3.753E-10 | NA | Li et al., 2019, Cheng et al., 2020 |
| Tryptophan | rs284191 | 1 | 92008636 | A | G | 0.6150 | -0.0060 | 0.0010 | 1.973E-09 | TGFBR3 | Li et al., 2019, Cheng et al., 2020 |
| Tryptophan | rs7463805 | 8 | 110822848 | T | C | 0.5213 | -0.0053 | 0.0009 | 4.596E-09 | NA | Li et al., 2019, Cheng et al., 2020 |
| Tryptophan | rs1559063 | 5 | 172281200 | C | G | 0.6209 | 0.0052 | 0.0009 | 7.818E-09 | ERGIC1 | Li et al., 2019, Cheng et al., 2020 |
| Tryptophan | rs38271 | 7 | 14273556 | A | G | 0.5925 | -0.0051 | 0.0009 | 1.185E-08 | DGKB | Li et al., 2019, Cheng et al., 2020 |
| Tryptophan | rs2111118 | 16 | 52606654 | T | C | 0.6207 | 0.0051 | 0.0009 | 1.212E-08 | FTO | Li et al., 2019, Cheng et al., 2020 |
| Tryptophan | rs603446 | 11 | 116159645 | T | C | 0.4448 | 0.0051 | 0.0009 | 1.383E-08 | ZPR1 | Li et al., 2019, Cheng et al., 2020 |
| Tryptophan | rs972459 | 3 | 193338191 | T | C | 0.5787 | 0.0050 | 0.0009 | 1.969E-08 | NA | Li et al., 2019, Cheng et al., 2020 |
| Tryptophan | rs4958379 | 5 | 153990234 | A | G | 0.4330 | -0.0050 | 0.0009 | 2.176E-08 | NA | Li et al., 2019, Cheng et al., 2020 |
| Tryptophan | rs1373962 | 5 | 83563618 | T | C | 0.5973 | 0.0050 | 0.0009 | 2.707E-08 | EDIL3 | Li et al., 2019, Cheng et al., 2020 |
| Tryptophan | rs9511152 | 13 | 18494767 | A | G | 0.5590 | -0.0050 | 0.0009 | 2.985E-08 | NA | Li et al., 2019, Cheng et al., 2020 |
| Tryptophan | rs710580 | 3 | 191291857 | A | C | 0.3566 | -0.0050 | 0.0009 | 3.574E-08 | P3H2 | Li et al., 2019, Cheng et al., 2020 |
| Tryptophan | rs7584842 | 2 | 11673711 | T | C | 0.5377 | -0.0050 | 0.0009 | 4.15E-08 | GREB1 | Li et al., 2019, Cheng et al., 2020 |
| Tryptophan | rs6480970 | 10 | 54238670 | A | G | 0.6159 | -0.0049 | 0.0009 | 4.287E-08 | LOC105378306/7 | Li et al., 2019, Cheng et al., 2020 |
| Tryptophan | rs4615256 | 5 | 62736623 | A | G | 0.5327 | 0.0049 | 0.0009 | 4.988E-08 | LOC107986418 | Li et al., 2019, Cheng et al., 2020 |
| Kynurenine | rs8051149 | 16 | 86436323 | A | G | 0.2127 | 0.0258 | 0.0025 | 9.073E-26 | SLC7A5 | Li et al., 2019, Cheng et al., 2020 |
| Kynurenine | rs3184504 | 12 | 110368991 | T | C | 0.4851 | 0.0149 | 0.0017 | 6.046E-18 | SH2B3 | Li et al., 2019, Cheng et al., 2020 |
| Kynurenine | rs10085935 | 8 | 39925424 | T | C | 0.3782 | -0.0103 | 0.0017 | 3.326E-09 | IDO2 | Li et al., 2019, Cheng et al., 2020 |
| Kynurenine | rs16924894 | 10 | 24885531 | A | T | 0.0240 | 0.0814 | 0.0146 | 2.329E-08 | NA | Li et al., 2019, Cheng et al., 2020 |
| Abbreviations: SNP, single-nucleotide polymorphism; Chr, chromosome; EAF, effect allel frequency; SE, standard error. | | | | | | | | | | | |

| Table S7. Baseline characteristics by quintiles of daily dietary tryptophan intake for participants from the NHANES Ⅲ^a^ | | | | | | |
| --- | --- | --- | --- | --- | --- | --- |
|  | Dietary tryptophan intake levels (g/d) | | | | |  |
|  | Quintile 1 | Quintile 2 | Quintile 3 | Quintile 4 | Quintile 5 | *P^c^* _trend_ |
|  | (≤0.5) | (0.6-07) | (0.8-0.9) | (1.0-1.2) | (≥1.3) |  |
| Number | 3917 | 3352 | 3080 | 3075 | 3254 | - |
| Age (y) | 46.06(44.76-47.35) | 46.30(45.11-47.49) | 44.19(42.81-45.57) | 42.36(41.32-43.40) | 37.86(37.01-38.72) | <0.001 |
| Body mass index^b^ (kg/m^2^) | 26.30(25.97-26.63) | 26.42(26.10-26.73) | 26.32(26.02-26.62) | 26.60(26.29-26.92) | 26.36(25.96-26.75) | 0.56 |
| Education (y) | 11.66(11.46-11.86) | 12.18(11.96-12.40) | 12.51(12.30-12.73) | 12.67(12.40-12.93) | 12.63(12.43-12.83) | <0.001 |
| Poverty Income Ratio | 2.71(2.55-2.87) | 3.02(2.88-3.17) | 3.26(3.05-3.46) | 3.26(3.12-3.40) | 3.09(2.89-3.29) | <0.001 |
| C-reactive protein (mg/dL) | 0.48(0.44-0.52) | 0.42(0.39-0.46) | 0.41(0.37-0.45) | 0.39(0.36-0.42) | 0.34(0.32-0.36) | <0.001 |
| Healthy Eating Index score | 58.66(57.95-59.36) | 64.96(64.24-65.67) | 66.60(65.76-67.44) | 65.80(64.96-66.64) | 61.96(61.16-62.77) | <0.001 |
| Gender |  | | | | | <0.001 |
| Male (%) | 223.8(21.9-25.9) | 36.1(33.9-38.4) | 43.0(39.9-46.2) | 55.4(52.9-57.9) | 76.6(74.3-78.8) |  |
| Female (%) | 76.2(74.1-78.1) | 63.9(61.6-66.1) | 57.0(53.8-60.1) | 44.6(42.1-47.1) | 23.4(21.2-25.7) |  |
| Race (%) |  | | | | | 0.003 |
| Non-Hispanic White | 73.7(70.1-76.9) | 76.6(73.2-79.6) | 77.8(74.7-80.7) | 77.9(75.0-80.6) | 73.8(70.9-76.5) |  |
| Non-Hispanic Black | 14.0(12.3-15.8) | 11.4(9.8-13.2) | 9.8(8.4-11.5) | 9.2(7.9-10.7) | 11.2(9.9-12.6) |  |
| Mexican-American | 5.0(4.1-6.0) | 4.5(3.7-5.5) | 5.5(4.6-6.6) | 5.2(4.3-6.1) | 6.0(5.0-7.2) |  |
| Other Race | 7.4(4.6-11.8) | 7.6(5.2-10.8) | 6.8(4.5-10.3) | 7.7(5.5-10.7) | 9.1(6.6-12.3) |  |
| Smoker (%) | 48.5(45.5-51.1) | 52.9(50.0-55.8) | 54.5(50.5-58.5) | 54.5(52.0-56.9) | 55.3(52.5-58.1) | 0.008 |
| Drinker (%) | 52.4(49.5-55.3) | 58.9(55.3-62.4) | 62.3(57.8-66.7) | 64.0(60.5-67.4) | 69.2(65.2-72.9) | <0.001 |
| Regular exercise (%) | 70.6(67.3-73.7) | 75.4(72.6-78.1) | 82.6(80.3-84.7) | 81.3(78.9-83.4) | 82.0(79.6-84.2) | <0.001 |
| Diabetes (%) | 5.6(4.8-6.6) | 5.9(5.1-6.9) | 5.2(4.2-6.4) | 5.2(4.2-6.4) | 4.1(2.9-5.6) | 0.134 |
| Hypertension (%) | 25.8(23.6-28.1) | 24.8(222.1-27.8) | 22.2(220.0-24.6) | 22.5(20.0-25.3) | 19.5(17.3-22.0) | 0.006 |
| Hypercholesterolemia (%) | 10.7(9.4-12.1) | 10.3(8.6-12.4) | 10.8(8.9-13.1) | 12.1(10.3-14.1) | 12.0(10.1-14.2) | 0.522 |
| Cancer (%) | 7.8(6.6-9.1) | 9.9(8.6-11.3) | 8.6(6.9-10.8) | 6.2(5.1-7.6) | 4.7(3.9-5.7) | <0.001 |
| Cancer mortality (%) | 7.2(6.0-8.5) | 7.5(6.2-9.1) | 6.5(5.2-8.1) | 6.1(5.1-7.3) | 4.9(4.0-6.0) | 0.033 |
| ^a^Values were weighted means(95% CI) or weighted percentages(95% CI). | | | | | | |
| ^b^Body mass index was calculated as weight in kilograms divided by height in meters squared. | | | | | | |
| ^c^P_trend_ was calculated by General linear models (continuous variables) or Pearson χ^2^ tests (bivariate relationships). | | | | | | |

| Table S8. Hazard ratios and 95% CIs for all-cancer mortality^a^ by quintiles of daily dietary tryptophan intake | | | | |
| --- | --- | --- | --- | --- |
|  | Cance mortality | Hazard ratio (95% CI) | | |
| Participants | (No./total No.) | Model 1^b^ | Model 2^c^ | Model 3^d^ |
| All participants (16678) |  | | | |
| Quintile 1(3254) | 199/3254 | 1(Ref.) | 1(Ref.) | 1(Ref.) |
| Quintile 2(3075) | 219/3075 | 1.004(0.739-1.365) | 1.066(0.788-1.442) | 1.151(0.847-1.563) |
| Quintile 3(3080) | 243/3080 | 0.997(0.703-1.413) | 0.994(0.703-1.406) | 1.078(0.757-1.533) |
| Quintile 4(3352) | 320/3352 | 1.137(0.825-1.567) | 1.163(0.856-1.581) | 1.205(0.881-1.648) |
| Quintile 5(3917) | 320/3917 | 1.154(0.834-1.597) | 1.233(0.942-1.612) | 1.160(0.872-1.545) |
| *P* _trend_ | - | 0.746 | 0.573 | 0.8 |
| Participants with cancer (1198) |  | | | |
| Quintile 1(152) | 26/152 | 1(Ref.) | 1(Ref.) | 1(Ref.) |
| Quintile 2(194) | 40/194 | 1.087(0.572-2.068) | 1.175(0.610-2.263) | 1.632(0.827-3.222) |
| Quintile 3(254) | 53/254 | 1.080(0.610-1.913) | 1.017(0.572-1.807) | 1.475(0.782-2.782) |
| Quintile 4(308) | 68/308 | 1.390(0.652-2.966) | 1.567(0.718-3.421) | 1.956(0.924-4.144) |
| Quintile 5(290) | 50/290 | 1.122(0.537-2.344) | 1.344(0.619-2.916) | 1.455(0.667-3.174) |
| *P* _trend_ | - | 0.803 | 0.576 | 0.448 |
| Non-cancer participants (15477) |  | | | |
| Quintile 1(3101) | 173/3101 | 1(Ref.) | 1(Ref.) | 1(Ref.) |
| Quintile 2(2881) | 179/2881 | 1.000(0.740-1.352) | 1.052(0.779-1.419) | 1.092(0.808-1.475) |
| Quintile 3(2826) | 190/2826 | 0.973(0.663-1.428) | 0.974(0.652-1.455) | 1.015(0.684-1.504) |
| Quintile 4(3043) | 252/3043 | 1.083(0.765-1.532) | 1.074(0.780-1.479) | 1.084(0.787-1.492) |
| Quintile 5(3626) | 270/3626 | 1.193(0.841-1.694) | 1.235(0.934-1.632) | 1.154(0.860-1.549) |
| *P* _trend_ | - | 0.68 | 0.63 | 0.908 |
| Kaplan-Meier test P | 0.085 | | | |
| ^a^Cance mortality was ascertained by the National Death Index(NDI). | | | | |
| ^b^Model 1 adjusted for age (continuous), gender (male, female) and race (Non-Hispanic White, Non-Hispanic Black, Mexican-American, Other race). | | | | |
| ^c^Model 2 adjusted for age (continuous), gender (male, female), race (Non-Hispanic White, Non-Hispanic Black, Mexican-American, Other race), education (continuous), poverty income ratio (continuous), smoker (yes, no), drinker (yes, no), regular exercise (yes, no). | | | | |
| ^d^Model 3 adjusted for age (continuous), gender (male, female), race (Non-Hispanic White, Non-Hispanic Black, Mexican-American, Other race), education (continuous), poverty income ratio (continuous), smoker (yes, no), drinker (yes, no), regular exercise (yes, no), healthy eating index score (continuous), C-reactive protein (continuous), diabetes (non-diabetic, diabetic). | | | | |
| Abbreviations: CI, confidence interval; No., number of events; ref., reference group. | | | | |
